# The Constitutive Lack of α7 Nicotinic Receptor Leads to Metabolic Disorders in Mouse

**DOI:** 10.3390/biom10071057

**Published:** 2020-07-16

**Authors:** Blandine Gausserès, Junjun Liu, Ewout Foppen, Cécile Tourrel-Cuzin, Ana Rodriguez Sanchez-Archidona, Etienne Delangre, Céline Cruciani-Guglielmacci, Stéphanie Pons, Uwe Maskos, Bernard Thorens, Christophe Magnan, Jamileh Movassat, Kamel Maouche

**Affiliations:** 1 Biology and Pathology of the Endocrine Pancreas, Université de Paris, BFA, UMR 8251, CNRS, F-75013 Paris, France; blandine.gausseres@wanadoo.fr (B.G.); ftdxfish@yahoo.fr (J.L.); cecile.tourrel-cuzin@u-paris.fr (C.T.-C.); delangre.e@icloud.com (E.D.); jamileh.movassat@paris7.jussieu.fr (J.M.); 2 Regulation of Glycemia by Central Nervous System, Université de Paris, BFA, UMR 8251, CNRS, F-75013 Paris, France; e.foppen@nin.knaw.nl (E.F.); celine.cruciani@gmail.com (C.C.-G.); christophe.magnan@univ-paris-diderot.fr (C.M.); 3 Center for Integrative Genomics, University of Lausanne, CH-1015 Lausanne, Switzerland; ana.rodriguez.1@unil.ch (A.R.S.-A.); bernard.thorens@unil.ch (B.T.); 4 Institut Pasteur, UMR 3571, CNRS, Department of Neuroscience, Integrative Neurobiology of Cholinergic Systems, CEDEX 15, 75724 Paris, France; stephanie.pons@pasteur.fr (S.P.); uwe.maskos@pasteur.fr (U.M.)

**Keywords:** α7 nAChR, β-cell mass, high glycemia, insulin resistance, fat mass

## Abstract

Objective: Type 2 diabetes (T2D) occurs by deterioration in pancreatic β-cell function and/or progressive loss of pancreatic β-cell mass under the context of insulin resistance. α7 nicotinic acetylcholine receptor (nAChR) may contribute to insulin sensitivity but its role in the pathogenesis of T2D remains undefined. We investigated whether the systemic lack of α7 nAChR was sufficient to impair glucose homeostasis. Methods: We used an α7 nAChR knock-out (α7^−/−^) mouse model fed a standard chow diet. The effects of the lack of α7 nAChR on islet mass, insulin secretion, glucose and insulin tolerance, body composition, and food behaviour were assessed in vivo and ex vivo experiments. Results: Young α7^−/−^ mice display a chronic mild high glycemia combined with an impaired glucose tolerance and a marked deficit in β-cell mass. In addition to these metabolic disorders, old mice developed adipose tissue inflammation, elevated plasma free fatty acid concentrations and presented glycolytic muscle insulin resistance in old mice. Finally, α7^−/−^ mice, fed a chow diet, exhibited a late-onset excessive gain in body weight through increased fat mass associated with higher food intake. Conclusion: Our work highlights the important role of α7 nAChR in glucose homeostasis. The constitutive lack of α7 nAChR suggests a novel pathway influencing the pathogenesis of T2D.

## 1. Introduction

Type 2 diabetes (TD2) is a major health problem that will affect 592 million people in the world by 2035 [1]. T2D is characterized by an inability to control glucose homeostasis in response to metabolic demand. The pathogenesis of T2D is mainly caused by insulin resistance and pancreatic β cell dysfunction [2]. In patients suffering pre-diabetes or diabetes, insulin resistance affects glucose uptake in response to insulin mainly in their skeletal muscle, liver and adipose tissue, leading to development of high glycemia [3]. T2D only occurs by deterioration in pancreatic β-cell function and/or progressive loss of pancreatic β-cell mass under the context of insulin resistance [4]. Thus, reduction of functional β-cell mass is a fundamental feature of T2D. Nevertheless, the factors promoting β-cell mass dysfunction in patients at different stage in their adult lives are poorly understood.

The release of acetylcholine (ACh), from parasympathetic vagal innervations of pancreatic islets, potentiates the glucose-stimulated insulin secretion [5] and is among several signals that modulate the β-cell proliferation [6]. ACh exerts its effects through muscarinic (mAChRs) and nicotinic acetylcholine receptors (nAChRs). The M3 mAChR subtype, widely expressed in pancreatic islets [7], is known as being the effector of cholinergic signaling in insulin secretion [8]. Nicotine is the exogenous agonist of nAChRs. Interestingly, experimental studies showed that nicotine impacts insulin secretion through nAChRs on β cell from isolated islets [9,10] and in pancreatic β cell line models [10,11]. These studies support clinical studies showing that chronic exposure to nicotine can lead to a reduction of β-cell function in smokers [12]. More recently, it has been reported that human polymorphisms, identified in the *Chrnb4* gene encoding for the β4 nicotinic subunit, can affect susceptibility to T2D [13]. Together, these observations suggest a contribution of nicotinic cholinergic signalling in insulin secretion. However, the mechanisms implying the nicotinic system in impaired insulin secretion are not yet determined.

Nicotinic receptors belong to a family of ionotropic receptor proteins formed by a dozen different nAChR subunit proteins [14]. These subunits are subdivided into α and β subfamilies, which form pentameric ion channels composed by either a single type of α subunit (homopentamers) or a combination of α and β subunits (heteropentamers) [15]. Pancreatic β cells express α3, α4, α5, α7, β2, and β4 subunits of nAChRs [9,10,11]. The homopentamer α7 nAChR is characterized by an elevated Ca^2+^ permeability [16] and has been involved in several important biological processes such as cell proliferation [17], apoptosis [18] and inflammation [19]. In humans, α7 nAChR expression in adipocytes has been negatively correlated with obesity [20]. On the other hand, pharmacological activation of α7 nAChR reduces body weight as well as food intake, and improves insulin sensitivity in *db/db* obese mice [21,22] by regulating inflammation in adipose tissue [23]. These results suggest a potential role of α7 nAChR in insulin sensitivity and in the pathogenesis of obesity. Interestingly, α7 nAChR is also expressed on pancreatic α cells [24]. However, the role of α7 nAChR in glucose homeostasis remains largely unknown.

Given that α7 nAChR is expressed in insulin target tissues, dysfunction of this receptor could affect glucose homeostasis. Here, we have addressed this issue by analyzing the systemic α7 nAChR knock-out in mice (α7^−/−^) fed a standard chow diet. We found that α7^−/−^ mice develop a metabolic sequence of events over time, which leads to a pre-diabetic state and a reduction in both α- and β-cell mass. Integrative analysis by combining a metabolic phenotyping study and transcriptome analysis in pancreatic islets from 6 mouse strains, including C57Bl6/J, the strain of α7^−/−^ mice, fed a high-fat diet, unveiled a significant and negative correlation between the expression of *chrna7* gene and fasting glycemia as well as basal insulinemia, validating our experimental data. Strikingly, old α7^−/−^ mice exhibited insulin resistance and a late-onset excessive gain in body weight through increased fat mass associated with higher food intake.

## 2. Experimental Procedures

### 2.1. Mice

Animal use and procedures were approved by the Ethics committee of the University of Paris Diderot (approval CEB-03-2016) and by the French Ministry of Research (approval 4189-2016012707493002v3). α7 nAChR^−/−^ (α7^−/−^), and Wild-Type (WT) C57BL/6J male mice (Charles River Laboratories, France) were housed in specific pathogen-free biosafety level 2 animal facility in a standard 12 h on/off light cycle (the lights are on from 8:20 PM to 8:20 AM), according to institutional guidelines. Mice were fed a standard chow diet for rodents (R03-10, Safe, France) and were provided with water and food ad libitum.

*Pregnant females*. Crosses were performed by caging a female (WT or α7^−/−^) with a male (WT or α7^−/−^) mouse for one night. The next morning, the presence of sperm in the vaginal smear was confirmed and this was taken as day 0.5 of pregnancy (E0.5). This female mouse was weighted and transferred to a separate cage. On day 18.5 of gestation (E18.5), pregnant mice (*WT*/*WT* and α*7^−/−^*/α*7^−/−^*) were weighted and their corresponding fetuses (f-*WT*/*WT* and f-α*7^−/−^*/α*7^−/−^*) were used in this study. Experiments were performed without previous fasting of the mothers. On day E18.5, maternal blood glycemia was measured from the tail vein. Then, the pregnant mice were anesthetized with pentobarbital (60 mg/kg).

*Fetuses*. Fetuses connected to the mother by their placenta and umbilical cord were successively exteriorized from the uterus, and weighted. Fetuses were euthanazied by decapitation and, after excision, whole fetal pancreases of f-*WT*/*WT* (*n* = 4–5) and f-α*7^−/−^*/α*7^−/−^* (*n* = 4–5) were immediately weighed and either stored at −80 °C for mRNA extraction either fixed in aqueous Bouin’s solution (1 h at room temperature), incubated in gelose, and embedded in paraffin. Each fetal pancreas was serially sectioned (5 µm) throughout its length and then mounted on slides for β- and α-cell immunohistochemistry and histomorphometry.

*Adults*. Adult male animals were killed by cervical dislocation at 10 and 25 weeks old.

**Scheme 1:**
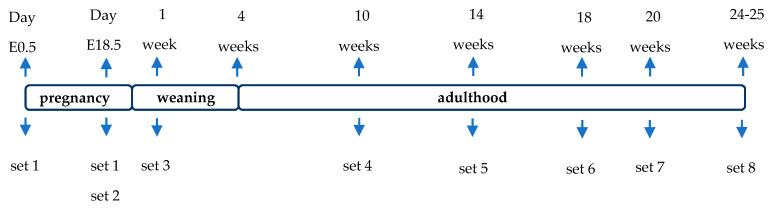
Schematic representation indicating the set of animals studied over time. Set 1 = pregnant mice; Set 2 = E18.5 fetuses; Set 3 = pups (1 week old); Set 4 = 10-weeks-old mice; Set 5 = 14-weeks-old mice; Set 6 = 18-weeks-old mice; Set 7 = 20-weeks-old mice and Set 8 = 24/25-weeks-old mice.

Different sets of mice, labeled set 1 to set 8, are depicted in Scheme 1 and assigned to the experiments described in Table 1. Half of the pregnant mice in set 1 were kept alive to collect 1-week-old mice.

### 2.2. Cell Culture

The rat insulinoma β-cell line, INS-1 832/13, was used between passages 20 and 40. Cells were cultured at 5% CO_2_ and 95% air at 37 °C in RPMI 1640 medium containing 11 mM d-glucose supplemented with 10% (vol/vol) heat-inactivated fetal bovine serum, 100 U/mL penicillin-streptomycin, 10 mM HEPES, 1 mM sodium pyruvate, 2 mM L-Glutamine and 50 µM beta-mercaptoethanol (Invitrogen, Saint Aubin, France).

The mouse adenoma α-cell line, α-TC1, was kindly provided by Y. Gosmain (Faculté de Médecine, Université de Genève, Genève, Switzerland) and was cultured in dulbecco’s modified eagle’s medium (11885084, ThermoFisher Scientific, Bleiswijk, Netherlands) supplemented with 10% fetal bovine serum (F7524, Sigma-Aldrich, Darmstadt, Germany), 15 mM HEPES (H0887, Merck, Darmstadt, Germany), 0.1 mM non-essentiel amino acids (1114050, ThermoFisher Scientific, Bleiswijk, The Netherlands), 0.02% bovine serum albumin (A8531, Merck, Darmstadt, Germany), 1.5 g/L sodium bicarbonate (27778.293, VWR, Fontenay-sous-Bois, France), and 2 g/L glucose (A2494001, ThermoFisher Scientific, Bleiswijk, The Netherlands).

### 2.3. Intraperitoneal Glucose, Insulin and Pyruvate Tolerance Tests

Glucose tolerance tests (GTT) were first performed in awake 10- and 25-weeks-old mice. GTT were performed by intraperitoneal injection of d-glucose (1.5 g/kg body weight, 24379.294, VWR, Fontenay-sous-Bois, France) in normal saline (0.9% NaCl) on overnight fasted mice. One week later, Insulin tolerance tests (ITT) were performed in these same mice by intraperitoneal injection of insulin (0.70 U/kg body weight; Actrapid, NovoNordisk, Bagsvaerd, Denmark) in normal saline (0.9% NaCl) after 6 h of food restriction. Similarly, Pyruvate tolerance tests (PTT) were performed in awake 25-weeks-old mice by intraperitoneal injection of sodium pyruvate (1 g/kg body weight; P2256, Sigma-Aldrich, Saint-Quentin Fallavier, France) in normal saline (0.9% NaCl) after 6 h of food restriction. Blood samples were collected from the tail vein for immediate glycemia measurement using a hand-held glucometer (Accu-Chek Performa, Roche, France). Glycemia was determined at 0, 15, 30, 45, 60, 90, and 120 min after glucose or insulin or pyruvate load. All AUC values were normalized as follows: AUC_normalized_ = AUC_total_—(the baseline value * 120).

### 2.4. In Vivo 2-Deoxyglucose Uptake Measurement

Twenty-five-week-old mice were fasted for 6 h and then were intraperitoneally injected with insulin at 0.70 U/kg body weight (Actrapid, NovoNordisk, Bagsvaerd, Denmark) and 2-deoxy-d-[1–14C] glucose (2DG) (5 μCi, Amersham). Blood was collected from the tail vein 0, 15, 30, 45, 90, and 120 min post injection. Animals were euthanized subsequently. Plasma d-[14C] 2DG concentration was determined from total blood after deproteinization with a Zn(OH)2 Ba(OH)2. Tissue d-[14C] 2DG and d-[14C] 2DG-6-phosphate content were determined as previously described [25]. Briefly, a piece of each tissue was weighed, dissolved in 1 M NaOH at 55 °C for 60–120 min, and then neutralized with 1 M HCl. d-[14C] 2DG and d-[14C] 2DG-6-phosphate were differentially precipitated by the use of a zinc hydroxide solution (0.3 M) or a perchloric acid solution (6%). Radioactivity was determined with a Packard Tri-Carb 460C liquid scintillation system.

### 2.5. Body Composition

Whole body composition (fat and lean mass) was measured in the morning using an Echo Medical Systems EchoMRI 100 (Whole Body Composition Analyzer, EchoMRI, Houston, TX, USA) in 18-, 20- and 23-weeks-old mice.

### 2.6. Food Intake and Body Weight Measurement

Food intake was measured from 14 until 24 weeks of age whereas body weight was measured between 18 and 23 weeks of age. For this, mice were housed individually and pre-weighed food (R03-10, Safe, France) was provided in standard stainless steel hoppers. Once a week, mice were briefly removed from their cages and weighed, and the amount of food remaining was measured to calculate the amount of food remaining. Intake was calculated as the weight (in grams) of food provided less that recovered. The result was divided by 7 to obtain average food intake per day per mouse (g/day/mouse).

### 2.7. Plasma Insulin Levels

On the day of IPGTT, plasma were obtained from whole blood collected (70 µL/sample) from the tail vein on heparin capillaries, then placed on ice, and centrifuged (3000 *g*, 5 min, 4 °C) to be directly frozen. Whole blood was collected at 0, 15, 60, and 120min in awake mice. Plasma insulin concentrations were measured at 0, 15, 60, and 120 min after glucose injection using ultra-sensitive mouse insulin ELISA kit (80-INSMSU-E01, Alpco, Eurobio, Les Ulis, France) according to the manufacturer’s protocol.

### 2.8. Pancreatic Insulin Content

Pancreatic tissues from 10-week-old α7^−/−^ and WT C57BL/6J mice stored at −20 °C in an acid-ethanol (1.5% HCl 12N, 75% ethanol, 23.5% distilled water) were homogenized with Polytron (PT 1600E, Kinematica AG, Littau-Lucerne, Switzerland) and centrifuged at 4 °C in the acid-ethanol solution (1.5% HCl 12N, 75% ethanol, 23.5% distilled water). Insulin concentrations in clarified supernatant were determined by using ultra-sensitive mouse insulin ELISA kit (80-INSMSU-E01, Alpco, Eurobio, Les Ulis, France) according to the manufacturer’s protocol.

### 2.9. Islet Isolation

Pancreata from 10-week-old α7^−/−^ and WT C57BL/6J mice were distended with a Hank’s solution containing liberase TL (5401020001, Roche, Mannheim, Germany), dissected free fat and digested in a 37 °C water bath for 17 min. Digestion was stopped by the addition of Hank’s solution supplemented with BSA and the pancreatic tissue was sedimented for 5 min. Undigested fragments and supernantant were carefully removed by vaccum. This step was repeated four times. After washing with Hank’s/BSA solution, islets were hand-picked under stereomicroscope and divided into different batches to be used for insulin secretion tests or to be stored at −80 °C for subsequent mRNA extraction.

### 2.10. Static Incubation for Insulin Test

After isolation, batches of 10 size-matched islets from α7^−/−^ and WT control mice were hand-picked and first maintained in Krebs-Ringer bicarbonate HEPES (KRBH) buffer (1 mL), containing 0.1% fatty-acid-free BSA and 2.8 mM glucose, in an incubator at 37 °C with humidified atmosphere and 5% CO_2_ for 1 h. Then batches of 10 size-matched islets were incubated for 1h at 37 °C in KRBH-0.1% BSA buffer (1 mL) containing 2.8 mM or 16.7 mM glucose. The supernatant (300 µL) was immediately collected and stored at −20 °C. Islets were centrifuged at room temperature (300 RPM, 1 min) and dry pellets were stored at −80 °C for DNA normalization assay. Insulin concentration was measured by using ultra-sensitive mouse insulin ELISA kit (80-INSMSU-E01, Alpco, Eurobio, Les Ulis, France).

### 2.11. Plasma Non-Esterified Fatty Acid Levels and Hepatic Glycogen Content

Plasma non-esterified fatty acids (NEFAs) levels were measured using the NEFA-HR (2) kit (Wako Diagnostics, Mountain View, CA, USA) according to the manufacturer’s protocol. For determination of hepatic glycogen content, livers from 25-week-old α7^−/−^ and WT control mice were collected, weighed and homogenized with Polytron (PT 1600E, Kinematica AG, Littau-Lucerne, Switzerland). Then, total hydrolysis of the glycogen into glucose was performed by using α-amyloglucosidase (10115-6G-F, Sigma-Aldrich, Saint-Quentin Fallavier, France) in a water-bath at 55 °C for 30 min. Glucose was precipitated by centrifugation at 4000 rpm for 5 min at 4 °C. The supernatants were collected and stored at −20 °C. Finally, the glucose concentration was determinated using a Glucose GOD-PAP assay kit (80009, Biolabo, Maizy, France) according to the manufacturer’s instructions.

### 2.12. Histomorphometric Studies

After dissection, whole pancreata from E18.5 fetuses were fixed in aqueous Bouin’s solution and embedded in paraffin. Each pancreatic block was serially sectioned (5 µm). For 10-week-old male mice, whole pancreata were carefully cut in half lengthwise to obtain two equal fragments. To avoid any bias due to differences in islet distribution or cell composition in the tail region of the pancreas compared to the head, each half consists of the head and tail of pancreata. Each fragment was weighted. One half was fixed in aqueous Bouin’s solution and embedded in paraffin. For each pancreas, 10 sections were randomly chosen at a fixed interval through the block, a procedure that has been shown to ensure that selected sections are representative of the entire pancreas [26]. The other half was homogenized and centrifuged at 4 °C in an acid-alcohol solution. The supernatant was stored at −20 °C until assayed. For histologic analysis, sections were stained only with eosin and hematoxylin while for morphometric analysis, sections were immunostained as previously described [26]. Briefly, sections were treated with hydrogen peroxide (3%) in TRIS-1X buffer for 15 min at room temperature to block endogenous peroxidase activity. After blocking in 10% goat serum, sections were incubated with rabbit anti-insulin (sc-8033, Santa-Cruz Biotechnology, Dallas, TX, USA) or rabbit anti-glucagon (K79bB10, Boster Biological Technology, Pleasanton, CA, USA) primary antibodies overnight at 4 °C. Then, sections were washed in TRIS-1X and incubated with an horseradish peroxidase conjugated goat anti-rabbit IgG (111-035-144, Jackson ImmunoResearch, Cambridge, UK) for 1 h at room temperature. The activity was revealed with 3,3′-diaminobenzidine-tetra-hydrochloride using a peroxidase substrate kit (DAB, Biosys-Vector, Compiègne, France). After staining, sections were mounted in Eukitt. Images were acquired on an Olympus BX60 microscope connected via a color video camera (XCD-U100CR, Sony, Tokyo, Japan) to a Compac PC computer. The relative β- or α-cell area in pancreatic sections and their respective cell mass were assessed by using Histolab software v.10.1 (Microvision instruments, Evry, France) as previously described [27]. For example, after insulin staining, the relative surface area of insulin-positive cells over the total pancreatic tissue was evaluated by morphometric method. The β-cell mass was then calculated by multiplying the relative β-cell fraction by the pancreas weight

### 2.13. RNA Extraction and Real-Time Quantitative RT-PCR

Different organs were harvested from mice that had never been tested in vivo. After dissection, RNA was extracted from fetal pancreata (E18.5), pancreatic islets of 10-weeks-old mice, liver of 10- and 25-weeks-old mice, muscle of 25-weeks-old mice, and adipose tissue of 10- and 25-weeks-old mice using the RNeasy mini kit (74104, Qiagen, Hilden, Germany) according to the manufacturer’s protocol. cDNA of each RNA sample was synthesized with M-MLV Reverse Transcriptase (28025013, Invitrogen, Saint Aubin, France) using random hexamer primers. The primers used for qPCR were designed from mouse sequences and using OLIGO primer analysis software v.7. The sequence of the primers used is provided in Table 2. Real-Time quantitative PCR (RT-qPCR) amplification reactions were carried out in a LightCycler 480 detection system (Roche Diagnostics, Mannheim, Germany) using LightCycler 480 SYBR Green I Master mix (04887352001, Roche Diagnostics, Mannheim, Germany) as previously described. mRNA transcript levels of four housekeeping genes (cyclo A, HPRT, TBP and RPL19) were assayed. On the base on the stability of the different genes, cyclo A was retained for normalization of transcripts in skeletal muscle and fetal pancreas, HPRT in αTC-1 cell line, TBP in adipose tissue and liver, as well as RPL19 in islets.

### 2.14. Correlation Studies in Mice Islets

Correlations between the expression of α7 nAChR in mouse islets and metabolic phenotypes were carried out on the basis of the database developed within the framework of the European programmes IMI Imidia and Rhapsody and according to a previously described methodology [28]. Briefly, male mice from six commonly used non-diabetic mouse strains were fed a high fat or regular chow diet for 3 months. Pancreatic islets were extracted and phenotypic measurements (basal glycemia and insulinemia, oral GTT, ITT) were performed at 2 days, 10 days, 30 days, and 90 days to assess diabetes progression. RNA-Seq was performed on islet tissue at each time-point and integrated with the phenotypic data in a network-based analysis [28].

### 2.15. Statistical Analysis

Statistical significance was determined between two groups by using the non-parametric two-tailed Mann–Whitney test, parametric two-tailed Multiple t tests and Two-Way Repeated Measures (RM) ANOVA from GraphPad Prism version 8.0 software (San Diego, CA, USA). A *p* value < 0.05 was considered statistically significant. Data are represented as mean ± SEM, and the number of cases is found in the figure legends.

## 3. Results

### 3.1. The Lack of α7 nAChR Induces a Chronic Mild High Glycemia in Mice Fed a Standard Chow Diet

**Figure 1:**
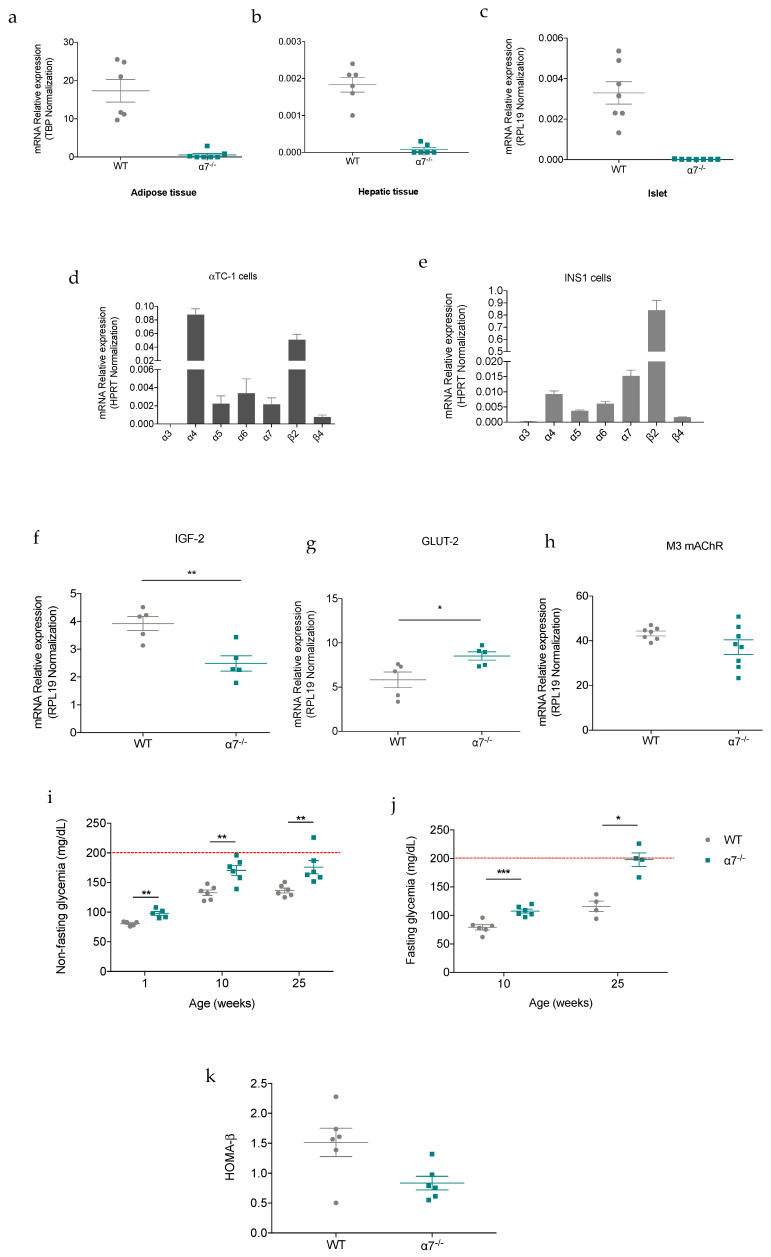
The constitutive lack of α7 nAChR induces a mild hyperglycemic state in male mice fed standard chow diet. mRNA expression of the α7 nicotinic subunit was measured in visceral adipose (**a**) and hepatic (**b**) tissues, and pancreatic islets (**c**). Gene expression levels were normalized to RPL19 (for islets) and TBP (for adipose and hepatic tissues) gene expression. Nicotinic subunits expressed in mouse α-TC1 (**d**; *n* = 5) and rat INS-1 832/13 cells (**e**; *n* = 5). Gene expression levels were normalized to HPRT gene expression. mRNA expression of IGF-2 (**f**), GLUT2 (**g**) and M3 mAChR (**h**) were measured from isolated islets from 10-week-old α7^−/−^ mice compared with age-matched WT controls. Gene expression levels were normalized to RPL19 gene expression. Non-fasting blood glucose level was measured in male mice at 1, 10 and 25 weeks old (**i**), and fasting glycemia were measured at 10 and 25 weeks old (**j**). Comparison of mean values of homeostatic model assessment for β-cell function (HOMA-β) index between 10-week-old α7^−/−^ and WT control (**k**). Each symbol represents a mouse (grey dots for WT mice and green squares for α7^−/−^ mice). All data are presented as mean ± SEM. Symbols *, ** and *** indicate a significant difference compared to control WT using unpaired Mann–Whitney two-tailed test (**f**–**h**,**k**; *n* = 5–7/group, * *p* < 0.05 and ** *p* < 0.01) and Multiple *t* tests (**i**,**j**, *n* = 5–6/group, * *p* < 0.05; ** *p* < 0.01; *** *p* < 0.001).

Using RT-qPCR, we found that mouse adipose tissue, liver, and pancreatic islets expressed the α7 nAChR (Figure 1a–c). In addition, α7 nAChR mRNA expression was detected in αTC-1 and INS1 cell lines, which are respectively pancreatic α- and β-cell models (Figure 1d,e). Interestingly, IGF-2 mRNA expression was significantly decreased in α7^−/−^ islets compared to control WT islets (Figure 1f), while GLUT2 glucose transporter mRNA expression was significantly increased (Figure 1g). However, M3 muscarinic acetylcholine receptor (mAChR) expression was not changed (Figure 1h). We measured non-fasting glycemia in α7^−/−^ and WT control mice at different ages. Non-fasting blood glucose concentration was significantly higher in α7^−/−^ mice compared with WT control mice (Figure 1i) from the beginning of the study (1 week of age), thus reflecting elevated glucose levels in response to the lack of α7 nAChR. Indeed, 10- and 25-week-old α7^−/−^ mice displayed glycemia reaching, respectively, a mean of 170.5 ± 8.21 and 175.83 ± 11.13 mg/dL, whereas age-matched WT control mice had mean values of 132.83 ± 4.61 and 136.5 ± 3.98 mg/dL. Although this difference was maintained until 25 weeks of age, non-fasting blood glucose levels in α7^−/−^ mice did not exceed 200 mg/dL, a glycemic value considered as diabetic in mice. Similarly, fasting glycemia (107.67 ± 3.54 mg/dL in α7^−/−^ mice versus 79.34 ± 4.54 mg/dL in WT mice) was significantly increased in 10-week-old α7^−/−^ mice when compared with age-matched WT control mice (Figure 1j). This difference has become more pronounced at 25 weeks of age (Figure 1j), reaching an average value of 197.75 ± 24.14 mg/dL in α7^−/−^ mice and 116,50 ± 9.30 mg/dL in WT control mice. Comparison of mean values of homeostatic model assessment for β-cell function (HOMA-β) index between 10-week-old α7^−/−^ and WT control mice indicates that α7^−/−^ β-cell function tends to be lower (Figure 1k). Together, these observations highlight that α7 nAChR deficiency leads to the development of a mild chronic hyperglycemic state, which is defined as a pre-diabetes [29].

### 3.2. Glucose metabolism Is Altered in α7^−/−^ mice Fed a Standard Chow Diet

**Figure 2:**
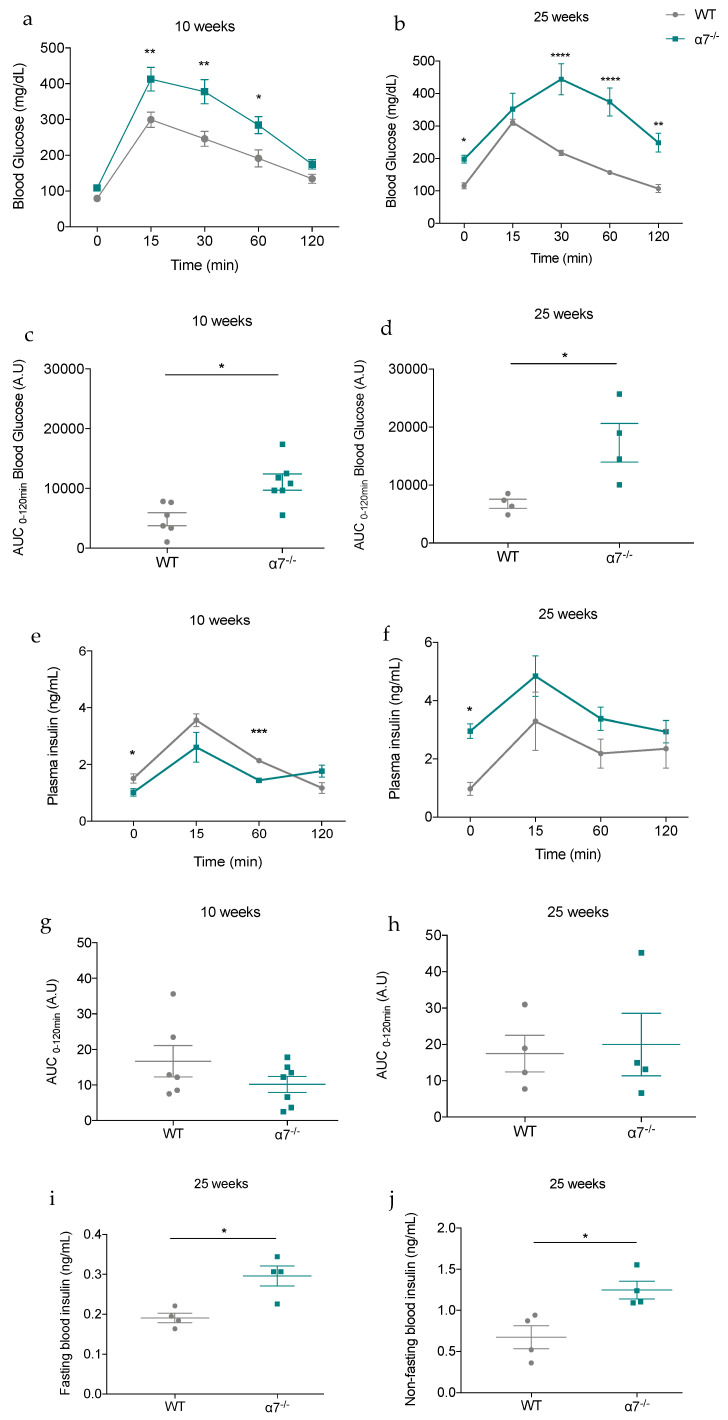
Glucose metabolism is impaired in α7^−/−^ mice fed a standard chow diet. All experiments in this figure were carried out in 10- and 25-week-old male WT control and α7^−/−^ mice. Glucose tolerance test was performed in overnight fasted mice with intraperitoneal injection of glucose (IPGTT) at 1.5 g/kg of body weight (**a**,**b**). Blood samples were taken at the time indicated from the tail vein of the same animals, and the area under the corresponding curve of blood glucose levels was calculated during 120min (**c**,**d**). Glucose-stimulated insulin secretion during IPGTT was analyzed at 0, 15, 60 and 120 min (**e**,**f**), and the area under the corresponding curve plasma insulin levels was calculated during 120min (**g**,**h**). Blood insulin levels under fed (**i**) and fasted (**j**) conditions in 25-week-old α7^−/−^ and WT control mice. Each dot represents a mouse (grey dots for WT mice and green squares for α7^−/−^ mice). All data are presented as mean ± SEM. Symbols *, **, *** and **** indicate a significant difference compared to control WT using Two-Way RM ANOVA (**a**,**b**,**e**,**f**; *n* = 6–7/group, * *p* < 0.05; ** *p* < 0.001; *** *p* < 0.0005; **** *p* < 0.0001) and unpaired Mann–whitney two-tailed test (**c**,**d**,**g**–**j**; *n* = 4–7/group, * *p* < 0.05). AUC, area under curve.

To explain chronic mild high glycemia observed in α7^−/−^ mice, we first performed a glucose tolerance test (GTT) to assess their ability to regulate glucose metabolism in response to an intraperitoneal injection of glucose. This test revealed significantly higher blood glucose concentrations after glucose administration in 10- and 25-week-old α7^−/−^ mice (Figure 2a,b, respectively) when compared with age-matched WT control mice. At 10 and 25 weeks of age, α7^−/−^ mice had impaired glucose tolerance, as shown by a significantly higher area under the blood glucose curve during the GTT (Figure 2c,d). To understand how glucose metabolism is disturbed in α7^−/−^ mice, we next evaluated glucose-stimulated insulin secretion (GSIS) in these mice. Our results showed that in vivo insulin secretion was significantly lower in 10-week-old α7^−/−^ mice at 0 and 60 min (Figure 2e) when compared with age-matched WT control mice. This could explain glucose intolerance and chronic mild high glycemia observed in Figure 1i and Figure 2a. However, there was no significant difference between areas under the curve (Figure 2g), suggesting an overall rate of insulin secretion, following an intraperitoneal glucose challenge, similar in both groups. Similarly, GSIS measured in 25-week-old α7^−/−^ mice was not different when compared with age-matched WT control mice (Figure 2f,h), except at 0 min. On the other hand, fed and fasting insulinemia were significantly higher in 25-week-old α7^−/−^ mice than WT control mice (Figure 2i,j). This observation suggests the development of insulin resistance, which could explain why basal glycemia rose in 25-week-old α7^−/−^ mice compared with 10-week-old α7^−/−^ mice (Figure 1j).

### 3.3. 25-Week-Old α7^−/−^ Mice Develop Insulin Resistance

**Figure 3:**
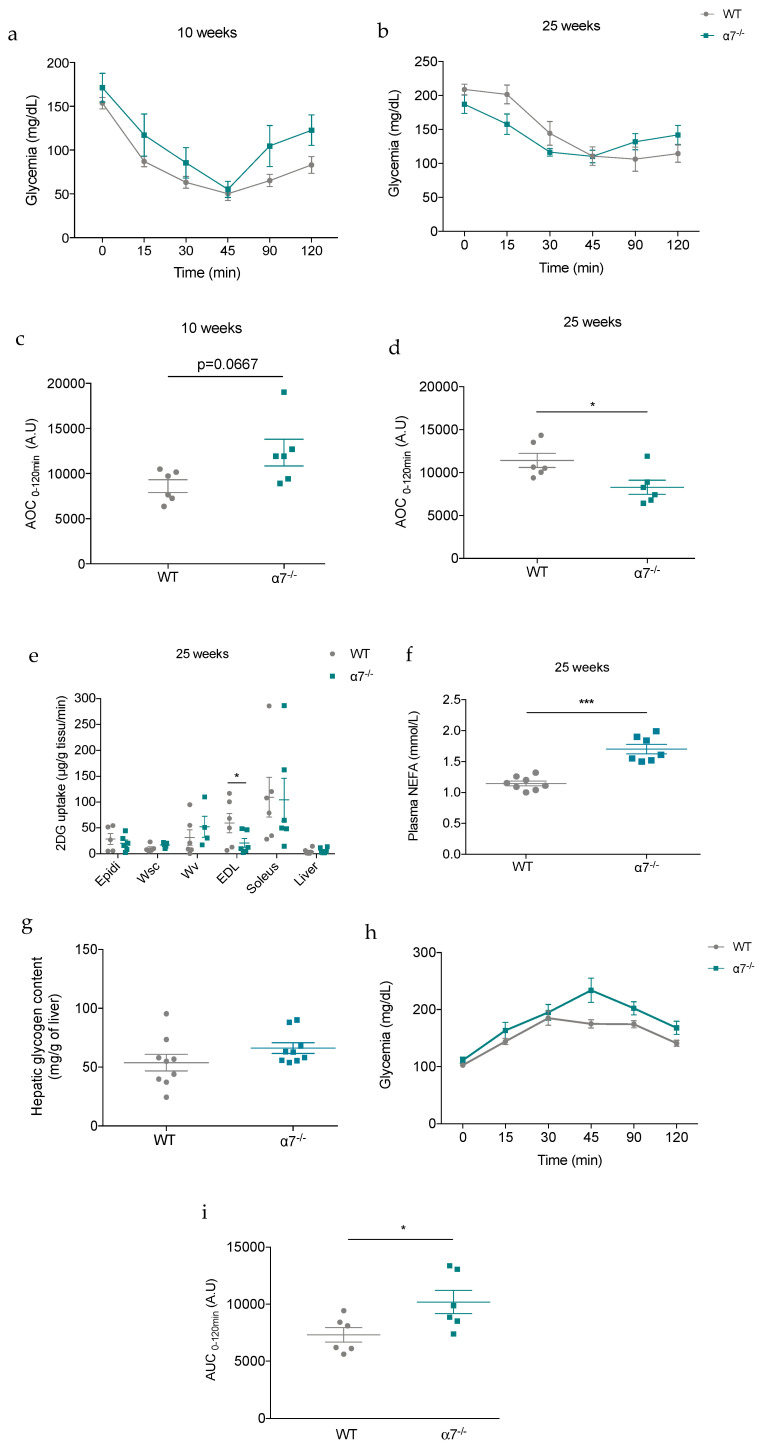
Twenty-five-weeks-old α7^−/−^ mice develop insulin resistance. All experiments in this figure were carried out in 10- and 25-week-old male WT control and α7^−/−^ mice. Insulin tolerance tests (0.75 U/kg) were performed in mice fasted 6h (**a**,**b**). Blood samples were taken at the time indicated from the tail vein of the same animals and the area over the curve of blood glucose levels was calculated during 120min (**c**,**d**). Insulin-stimulated 2DG uptake in epididymal (Epidi), subcutaneous (Wsc) and visceral (Wv) adipose tissues, livers, and soleus as well as EDL muscles from 25-week-old WT control and α7^−/−^ mice (**e**). Comparison of plasma NEFA concentration from 25-week-old WT control and α7^−/−^ mice (**f**) and of hepatic glycogen content (**g**). Blood glycemia was measured following intraperitoneal injection of 1g/kg sodium pyruvate (**h**) and the area under the corresponding curve was calculated (**i**). Each dot represents a mouse (grey dots for WT mice and green squares for α7^−/−^ mice). All data are presented as mean ± SEM. Symbols * and *** indicate a significant difference compared to control WT using unpaired Mann–Whitney two-tailed test (**c**,**d**,**f–h** *n* = 4–9/group, * *p* < 0.05; *** *p* < 0.001) and Multiple t tests (e, *n* = 5–7/group, * *p* < 0.05). AOC, area over curve.

*Impact on insulin sensitivity.* We then performed an intraperitoneal insulin tolerance test (ITT). Surprisingly, the results revealed a strong trend (*p* = 0.0667) towards increased insulin sensitivity in 10-week-old α7^−/−^ mice compared with age-matched WT control mice (Figure 3a). Although this difference was also not statistically significant (Figure 3c), these observations indicate that 10-week-old α7^−/−^ mice seem to be more insulin sensitive than WT controls, likely to compensate for insulin secretion as previously described in rats with mild insulin deficiency induced by neonatal streptozotocin [30]. The course of the curve in 10-week-old α7^−/−^ mice seems to increase between 45 and 120 min compared to WT control mice, suggesting probably a glucagon counterregulatory response to prevent further glucose reduction. As expected, 25-week-old α7^−/−^ mice revealed an insulin resistance during insulin tolerance test (Figure 3b), as shown by a significantly higher area over the curve compared with those of WT control mice (Figure 3d).

*Impact on insulin target tissues*. Because insulin resistance is defined as an inability of peripheral tissues, such as the skeletal muscle, liver, and adipose tissue, to increase glucose uptake in response to insulin, we further investigated which of these tissues had become insulin resistant in 25-week-old α7^−/−^ mice. Measurements of radiolabeled 2-deoxyglucose uptake, a glucose analog, allowed us to show that only insulin-stimulated glucose uptake in the EDL skeletal muscle was significantly reduced in 25-week-old α7^−/−^ mice as compared with age-matched WT mice (Figure 3e). It is well established that elevated plasma non-esterified fatty acid (NEFA) levels decrease insulin-stimulated glucose uptake in the skeletal muscle [31,32]. We subsequently quantified plasma NEFA concentrations. The analysis showed that plasma NEFA levels in 25-week-old α7^−/−^ mice were significantly higher than those of age-matched WT mice (Figure 3f).

*Impact on neoglucogenesis*. Since elevated circulating NEFA levels have direct effects on hepatic glucose stores as well as on neoglucogenesis [33,34], we then wanted to investigate whether 25-week-old α7^−/−^ mice could display an impaired hepatic glucose homeostasis. We found that absence of α7 nAChR had no effect on hepatic glycogen content in mice (Figure 3g). However, when mice were challenged with pyruvate, a gluconeogenic precursor, the lack of α7 nAChR impaired pyruvate tolerance as represented by a significant increase in AUC (Figure 3h,i). It is important to note that glycemia was very similar during 30 min following the pyruvate bolus injection in both groups (Figure 3h), reflecting an unaltered glucose production in α7^−/−^ mice. Nevertheless, unlike WT control mice, blood glucose levels in α7^−/−^ mice continued to rise until 45 min after injection and then progressively decreased (Figure 3h). As glucose uptake was reduced in α7^−/−^ mice, the clearance of glucose from gluconeogenesis was delayed, thus explaining the significant increase in AUC observed in α7^−/−^ mice (Figure 3i). Therefore, this effect reflects insulin resistance rather than hepatic dysfunction in 25-week-old α7^−/−^ mice. Taken together, these results show that glucose dyshomeostasis observed in α7^−/−^ mice worsened over time.

### 3.4. α7 nAChR Deficiency Is Associated to a Reduction in β-cell Mass in Mice

**Figure 4:**
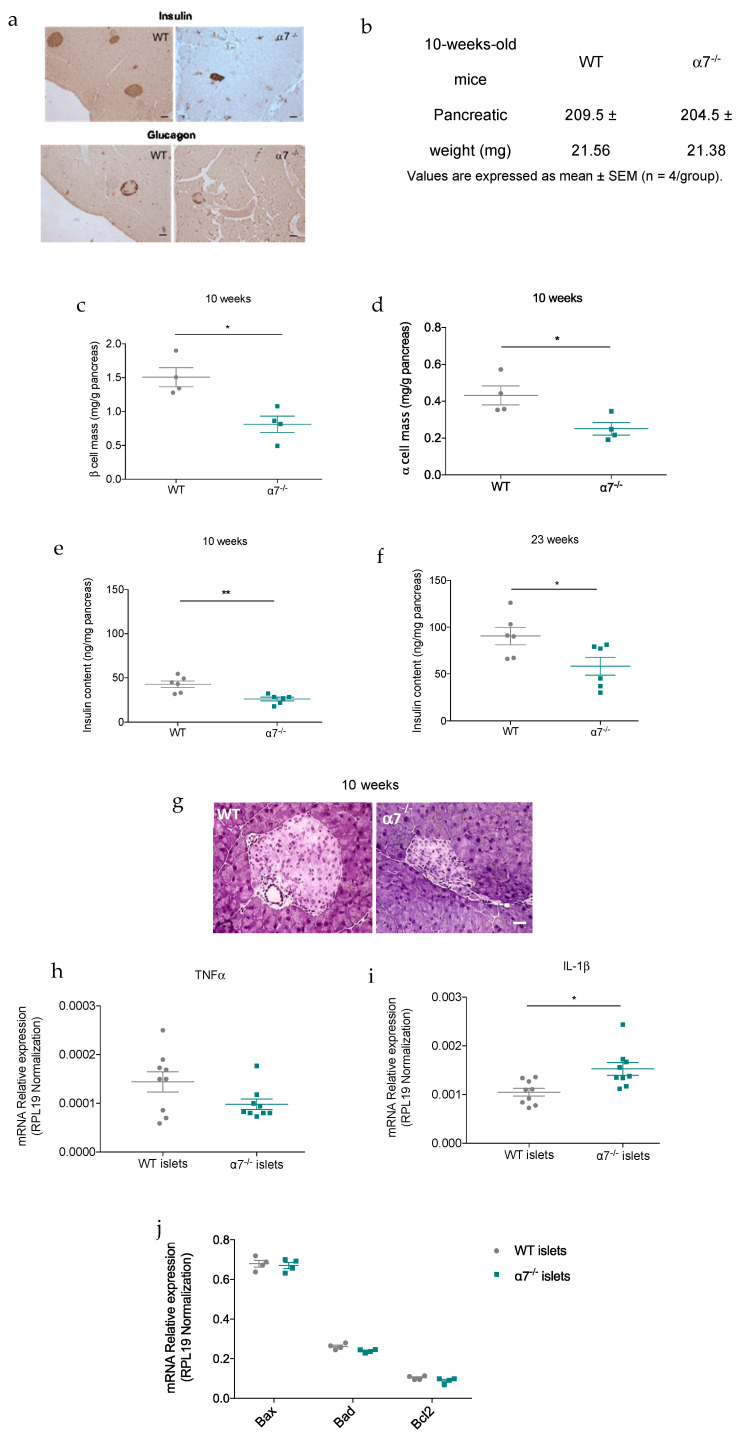
α7 nAChR deficiency is associated with a reduction in β-cell mass in mice. Pancreata were harvested from 10-week-old male α7^−/−^ and WT control mice. α-and β-cell mass were measured by morphometric analysis after glucagon or insulin immunostaining. Illustrative images of insulin and glucagon immunostaining from a WT control (left) and α7^−/−^ (right) mouse at 10 weeks of age (Scale bar = 1 mm) (**a**), pancreas weight (**b**), β-cell mass (**c**), and α-cell (**d**) per pancreas of four animals in each group at 10 weeks of age. Insulin content per pancreas was significantly reduced in 10- and 23-weeks-old α7^−/−^ mice compared with WT controls (**e**,**f**). Representative hematoxylin stained pancreatic section from a WT control (left) and α7^−/−^ (right) mouse at 10 weeks of age (Scale bar = 30µm) (**g**). mRNA expression of characteristic pro-inflammatory cytokines (TNFα and IL-1β) (**h**,**i**) and pro-apoptosis markers as Bax, Bad and Bcl2 (**j**) were measured from isolated islets from 10-week-old α7^−/−^ mice compared with age-matched WT controls. Gene expression levels were normalized to RPL19 (for inflammatory cytokines) and HPRT (for apoptosis markers) gene expression. Each dot represents a mouse (grey dots for WT mice and green squares for α7^−/−^ mice). All data are presented as mean ± SEM. Symbols * and ** indicate a significant difference compared to control WT using unpaired Mann–Whitney two-tailed test (**c**–**f**,**h**–**j**, *n* = 4–9/group, * *p* < 0.05; ** *p* < 0.005) and Multiple t tests (**j**, *n* = 4/group).

Given that fed and fasting glycemia were significantly higher in 10-week-old α7^−/−^ mice, we investigated the effects of α7 nAChR deficit on pancreatic islet mass in 10-week-old mice. Interestingly, morphometric measurements showed that β- and α-cell mass were significantly reduced in α7^−/−^ mice compared with WT control mice by approximately 48% (Figure 4a–c) and 46% (Figure 4a,b,d), respectively. Moreover, pancreatic insulin content was also significantly decreased in 10- and 25-week-old α7^−/−^ mice (Figure 4e,f), suggesting that β-cell mass is still reduced in 25-week-old α7^−/−^ mice. Since it has been reported that the cholinergic anti-inflammatory pathway was regulated by α7 nAChR [19], we then asked if significant reduction in pancreatic islet mass identified in 10-week-old α7^−/−^ mice was caused by islet inflammation. Hematoxylin and eosin staining of pancreatic sections from 10-week-old α7^−/−^ mice revealed neither inflammation nor islet fibrosis (Figure 4g). Moreover, mRNA expression quantification by RT-qPCR from isolated α7^−/−^ islets indicated that apoptotic marker and pro-inflammatory cytokine levels were not different compared to WT control islets (Figure 4h–j), confirming the absence of pancreatic inflammation in 10-week-old α7^−/−^ mice. Overall, these data suggest that impaired glucose homeostasis in α7^−/−^ mice is related to a reduced islet mass, which is not a secondary consequence of inflammation, thus highlighting a novel role for α7 nAChR in islet mass homeostasis.

### 3.5. α7 nAChR Deficiency Is Associated to Embryonic Remodeling of Pancreatic Islet Mass

**Figure 5:**
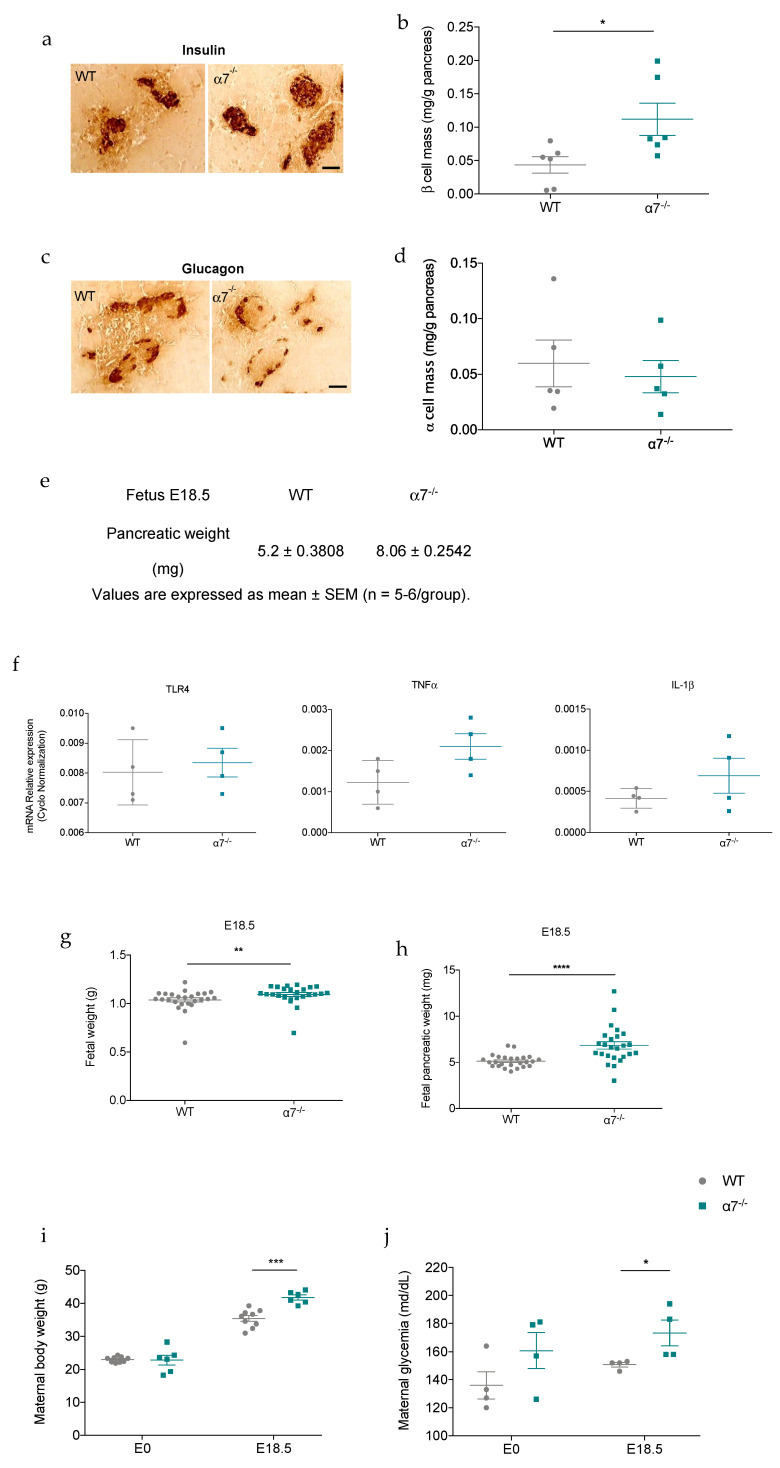
α7 nAChR deficiency is associated to embryonic remodeling of pancreatic islet mass. Pancreata were harvested from α7^−/−^ and WT control fetuses at E18.5, and α- and β-cell mass were measured by morphometric analysis after glucagon or insulin immunostaining. Illustrative images of insulin (**a**) or glucagon (**c**) staining in pancreas from a WT control (left) and α7^−/−^ (right) fetus at E18.5 (Scale bare = 30 µm). β-cell mass (**b**,**d**) α-cell mass in pancreatic sections of α7^−/−^ fetuses at E18.5 are shown compared with WT controls. Pancreas fetal weight used to quantify β- and α-cell mass are expressed as mean ± SEM (**e**). At E18.5, fetal pancreas were analyzed for mRNA expression of pro-inflammatory markers such as TLR4, TNFα and IL-1β (**f**). Gene expression levels were normalized to Cyclophilin A gene expression. Body (**g**) and pancreas (**h**) weight were measured in α7^−/−^ and WT control fetuses at embryonic day 18.5 (E18.5). Maternal weight (**i**) and glycemia (**j**) were measured the morning on which a vaginal plug (E0.5) was present and at day 18.5 (E18.5) of the pregnancy. Each dot represents a mouse (grey dots for WT mice and green squares for α7^−/−^ mice). All data are presented as mean ± SEM. Symbols *, **, *** and **** indicate a significant difference compared to control WT using unpaired Mann–Whitney two-tailed test (**b**,**d**,**f–h**, *n* = 4–25/group, * *p* < 0.05; ** *p* < 0.01; *** *p* < 0.001 and **** *p* < 0.0001) and Multiple t tests (**i**,**j**, *n* = 4–9/group, * *p* < 0.05, *** *p* < 0.001).

Since we observed that the lack of α7 nAChR induced a pronounced reduction of islet mass, higher non-fasting blood glucose levels in 1-week-old α7^−/−^ mice led us to measure islet mass at embryonic day 18.5 (E18.5) and to investigate whether α7^−/−^ mice were born with a deficit of islet mass. Unlike what we have observed in adulthood, β-cell mass (Figure 5a,b,e) was significantly higher in E18.5 α7^−/−^ fetuses than E18.5 WT control fetuses. In contrast, α-cell mass was similar in both groups (Figure 5c–e). In addition, there was no difference in pro-inflammatory cytokine expression in both groups (Figure 5f), thereby reinforcing the idea that α7 nAChR could be involved in islet mass homeostasis. It is important to note that body and pancreas weight from E18.5 α7^−/−^ fetuses were significantly higher than those from WT fetus (Figure 5g,h), suggesting that these fetuses are macrosomic. Measurements of maternal weight and glycemia at day 18.5 of the pregnancy revealed that α7^−/−^ pregnant mothers exhibit an mild hyperglycemic intrauterine environment (Figure 5i,j).

### 3.6. Body Weight and Food Intake Are Disturbed in α7^−/−^ Mice Fed a standard Chow Diet

**Figure 6:**
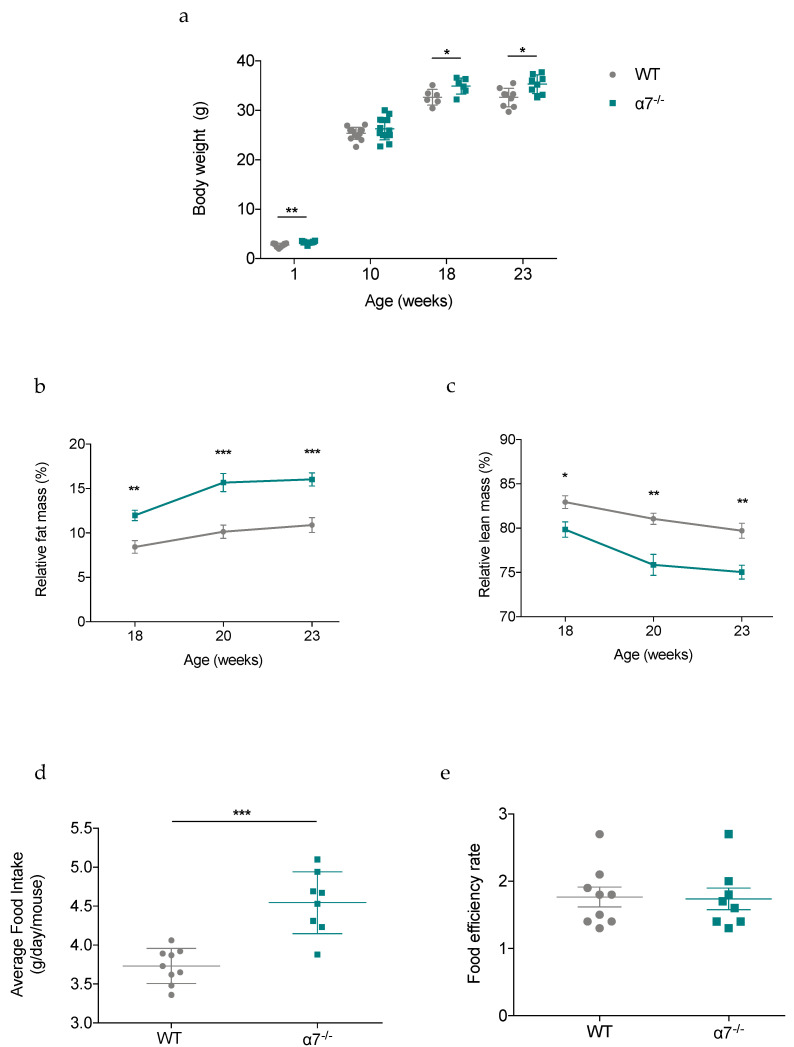
Body weight and food intake are disturbed in α7^−/−^ mice fed a standard chow diet. Body weight was measured in WT control and α7^−/−^ mice at 1, 10, 18, and 23 weeks of age (**a**). The percentage of fat (**b**) or lean (**c**) mass relative to body weight was assessed by EchoMRI in 18-, 20- and 23-week-old in WT control and α7^−/−^ mice. Cumulative average daily food intake between 14 and 24 weeks of age in α7^−/−^ mice compared with age-matched WT controls (**d**). Evaluation of food efficiency rate was calculated as weight gain (g/day) over food intake (g/day) from 14 to 24 weeks old in α7^−/−^ and WT control mice (**e**). Each dot represents a mouse (grey dots for WT mice and green squares for α7^−/−^ mice). All data are presented as mean ± SEM. Symbols *, ** and *** indicate a significant difference compared to control WT using unpaired Mann–whitney two-tailed test (**a**–**e**, *n* = 4–13/group, * *p* < 0.05; ** *p*< 0.01 and *** *p* < 0.001).

Literature reports that increased body weight and adiposity are hallmarks of insulin resistance [35]. Given that α7^−/−^ mice develop insulin resistance over time, we evaluated the progression of their body weight with age. As expected, body weight was significantly increased in 18- and 23-week-old α7^−/−^ mice compared with age-matched WT control mice (Figure 6a). Besides, body weight at 10 weeks of age was similar (25.34 ± 0.34 g for WT group versus 26.29 ± 0.62 g for α7^−/−^ group, *p* > 0.3) in both groups (Figure 6a). Higher body weight (2.74 ± 0.12 g for WT group versus 3.30 ± 0.08 g for α7^−/−^ group, *p* < 0.002) seen in 1-week-old α7^−/−^ pups (Figure 6a) is likely a consequence of mild hyperglycemic intrauterine environment (Figure 5h,j), as previously described [36]. This is agreed with higher body and pancreatic fetal weight in E18.5 α7^−/−^ fetuses (Figure 5f,g), reflecting a macrosomic phenotype that has been maintained during at least the first postnatal week and has returned to normal weight at 10 weeks of age (Figure 6a). Given their more important growth rate, assessment of body composition changes was performed in 18-, 20- and 23-week-old mice. Strikingly, the percentage of relative fat mass at the different ages showed that α7^−/−^ mice gained more weight as fat mass than WT control mice (Figure 6b). More importantly, fat mass gain between weeks 18 and 23 was more pronounced in α7^−/−^ mice (Figure 6b). On the other hand, 18-, 20- and 23-week-old α7^−/−^ mice lost significantly more lean mass compared with age-matched WT control mice (Figure 6c). This difference was also more marked between weeks 18 and 23 in α7^−/−^ mice than in WT control mice (Figure 6c). In addition, α7^−/−^ mice exhibited a disturbed food behavior as shown by average food intake significantly higher than WT control mice between weeks 18 and 23 (Figure 6d). However, their food efficiency was not different as compared to WT control mice (Figure 6e), indicating that food assimilation did not contribute to body weight gain. In summary, insulin-resistant α7^−/−^ mice increased their body weight due to their higher food consumption, which led to an increase in fat mass, a known factor to contribute in the pathogenesis of T2D.

### 3.7. Adipose Tissue in 25-Week-Old α7^−/−^ Mice Exhibits Higher Expression of Pro-Inflammtory Genes

**Figure 7:**
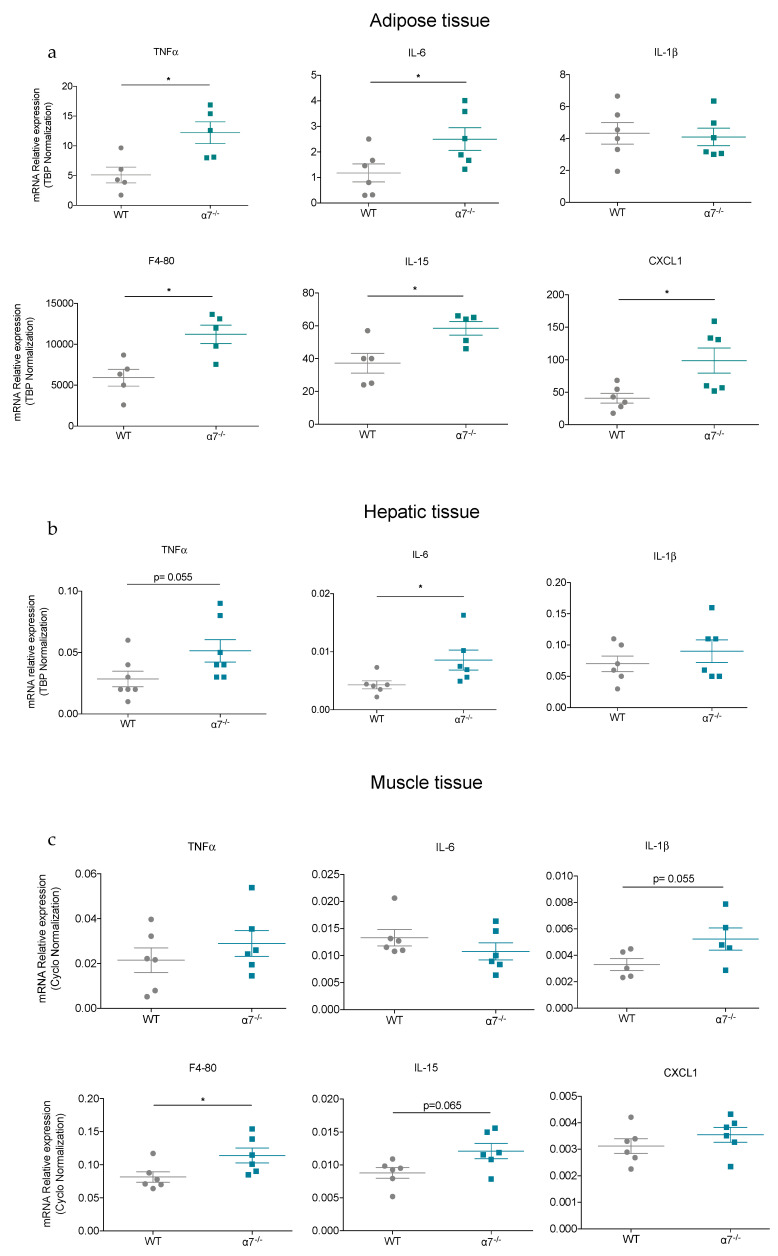
Twenty-five-week-old α7^−/−^ mice develop adipose inflammation. mRNA expression of characteristic pro-inflammatory marker TNFα, IL-6, IL-1β F4/80, cytokine IL-15 and chemokines CXCL1 were measured in visceral adipose (**a**), in hepatic (**b**) and muscle tissues (**c**) from 25-week-old α7^−/−^ mice compared with age-matched WT controls. Gene expression levels were normalized to TBP (for adipose and hepatic tissues) and Cyclophilin A (for muscle tissue) gene expression. Each dot represents a mouse (grey dots for WT mice and green squares for α7^−/−^ mice). All data are presented as mean ± SEM. Symbol * indicates a significant difference compared to control WT using unpaired Mann–Whitney two-tailed test (**a**–**c**, *n* = 5–6/group, * *p* < 0.05).

We showed increased body weight gain and adiposity in old α7^−/−^ mice. As it is now well established that inflammation, and particularly in adipose tissue, is a hallmark of insulin resistance and obesity [37,38], we thus reasoned that inflammation also could occur in adipose and peripheral tissues and be associated with the overweight and insulin-resistant phenotype we observed. We studied this possibility by measuring pro-inflammatory gene expression such as TNFα, IL-1β and IL-6. As shown in Figure 7, adipose tissue in 25-week-old α7^−/−^ mice had significantly higher expression of genes encoding for TNFα, IL-6, F4/80 and IL-15 as well as CXCL1 (Figure 7a), which are associated with obesity [39,40]. IL-6 mRNA expression level was significantly enhanced in liver tissue whereas TNFα mRNA expression markedly trended to be higher (*p* = 0.055) in 25-week-old α7^−/−^ mice than age-matched WT mice (Figure 7b). However, expression of IL-1β gene, in adipose tissue (Figure 7a) and liver (Figure 7b), was similar in both groups of mice. In muscle tissue, IL-1β and F4/80 mRNA expression were significantly increased from 25-week-old α7^−/−^ mice compared with age-matched WT control mice (Figure 7c), whereas IL-15 mRNA expression tended to be higher (*p* = 0.065) in 25-week-old α7^−/−^ mice (Figure 7c). We concluded that levels of pro-inflammatory markers were much higher in adipose tissue than muscle tissue and liver in 25-week-old α7^−/−^ mice, supporting an association of insulin resistance and body weight gain with increased inflammation.

### 3.8. Correlation between the Expression of α7 nAChR in Mouse Islets and Fasting Glycemia and Insulinemia

**Figure 8:**
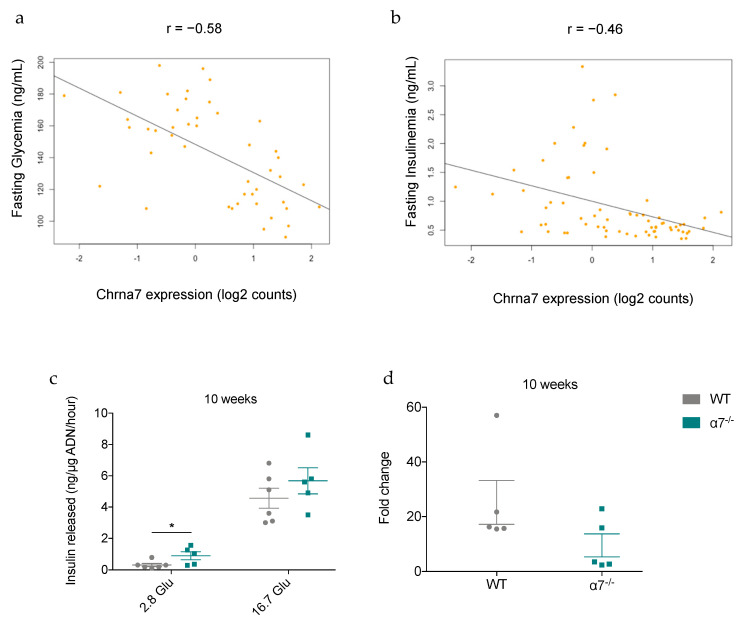
Correlation between the expression of α7 nAChR in mouse islets and fasting glycemia and insulinemia. Scatter plots showing that *chrna7* gene expression, encoding for the α7 nicotinic subunit, from islets is negatively correlated with fasting glycemia (**a**); Spearman’s r = −0.58, *p*-value = 1.7 × 10^−5^ (all samples) and with fasting insulinemia (**b**); Spearman’s r = −0.46, *p* value = 0.001 (all samples). Glucose-induced insulin secretion was measured in isolated islets from 10-week-old α7^−/−^ and WT control mice (**c**). Insulin released at 2.8 mM and 16.7 mM glucose was determined as described in Experimental procedures. The fold change in insulin secretion in response to 16.7 mM glucose was measured after 1-h static incubations (**d**). Each dot represents a mouse (grey dots for WT mice and green squares for α7^−/−^ mice). All data are presented as mean ± SEM. Symbol * indicates a significant difference compared to control WT using unpaired Mann–Whitney two tailed test (**c**,**d**, *n* = 5–6/group, * *p* < 0.05). 2.8Glu: 2.8 mM glucose; 16.7Glu: 16.7 mM glucose.

In parallel, metabolic phenotyping measurements in high-fat-fed male mice from six commonly used non-diabetic mouse strains, including C57Bl6/J strain, were recorded at 2 days, 10 days, 30 days, and 90 days to evaluate diabetes progression. To identify metabolic phenotype-related genes, RNA sequencing was performed on pancreatic islets from these mice, which express α7 nAChR. Transcriptomic analysis was performed at each time-point and integrated with the phenotypic data. Our results identified *Chrna7* gene, encoding for the α7 subunit of nAChR, as being one of several genes related to fasting glycemia and fasting insulinemia (Figure 8a,b). Its expression in islets was significantly and negatively correlated with fasting glycemia (r = −0.58, p value = 1.7 × 10^−5^) (Figure 8a) and fasting insulinemia (r = −0.46; *p* = 0.001) (Figure 8b), thus confirming our results observed in 25-week-old α7^−/−^ nAChR mice (Figure 1j and Figure 2i). Next, we studied insulin secretion, in freshly isolated islets from 10-week-old mice, in response to 2.8 mM and 16.7 mM Glucose. In agreement with our transcriptomic data, we found that basal insulin secretion in 2.8 mM glucose was significantly increased by approximately 3-fold in freshly isolated α7^−/−^ islets when compared with WT controls (Figure 8c). However, the difference was not significant at 16.7 mM Glucose (Figure 8c). The fold change by glucose at 16.7 mM over that at 2.8 mM glucose was not statistically significant but exhibited a trend for decrease in isolated α7^−/−^ islets (Figure 8d), indicating the onset of deterioration of β-cell function.

## 4. Discussion

The present study was undertaken to examine the role of α7 nAChR in glucose homeostasis. In contrast to previous studies that employed pharmacological approaches, we have here used transgenic mice lacking the α7 nAChR and fed a normal chow diet to address this question. Evaluation of glucose homeostasis revealed that 1/ the lack of α7 nAChR in 10-week-old mice leads to a reduced β-cell mass and chronic mild high glycemia without islet inflammation; 2/10-week-old α7^−/−^ mice exhibit impaired glucose tolerance and a tendency for higher insulin sensitivity, whereas 25-week-old α7^−/−^ mice showed both glucose intolerance and skeletal muscle insulin resistance, and 3/10 to 25-week-old α7^−/−^ mice gained more weight through a marked increase of fat mass associated with higher food intake. Together, our findings provide several lines of evidence that the lack of α7 nAChR is a significant factor in the pathogenesis of chronic high glycemia and suggest that α7 nAChR contributes to regulate glucose homeostasis.

In T2D, reduction in β-cell mass is a consequence of excessive apoptosis and/or inadequate proliferation (through replication and/or neogenesis) of β-cells [41,42]. It has been demonstrated that the pancreatic branch of the vagus nerve releases acetylcholine (ACh) to regulate β-cell secretory function [43], β-cell proliferation [6,44,45], and survival signals [46,47]. The effects of ACh are mediated through muscarinic (mAChR) and nicotinic receptors. Although M3 mAChR is the main subtype expressed in β-cell [48], its absence in mice did not change β-cell mass [49] and the lack of α7 nAChR did not modify its expression (Figure 1h), indicating that M3 mAChR is not involved in the regulation of β-cell mass. Here, we found that β-cell mass was increased in E18.5 α7^−/−^ fetuses and decreased in 10-week-old α7^−/−^ mice, reflecting thus the progressive loss of β-cell mass in response to the deletion of α7 nAChR in islets. On the other hand, β-cell α7 nAChR stimulation, in the multiple low-dose streptozotocin-induced mouse T1D model, preserved β-cell mass by increasing survival signals [46]. However, our findings showed that pancreata from α7^−/−^ fetuses or islets from 10-week-old α7^−/−^ mice displayed no evidence of apoptosis or inflammation (Figure 4h–j and Figure 5f), suggesting instead a defective β-cell proliferation. Moreover, over-expression of IGF-2, specifically in β-cells, led to an increased β-cell mass in transgenic mice [50], whereas a reduced pancreatic expression of IGF-2 was associated to a decreased β-cell mass [51]. Since islets from 10-week-old α7^−/−^ mice (Figure 1f) exhibit a significant reduction of IGF-2 mRNA expression, one could speculate that the lack of α7 nAChR in β-cell associated to downregulation of IGF-2 mRNA levels could, among other factors, contribute to reduce β-cell mass. Future studies are required to determine if there is a relationship between α7 nAChR and IGF-2. We found that α7^−/−^ mice exhibited a reduction of α- and β-cell mass. Whether the α7 nAChR dysfunction is a result of an impairment of replication, neogenesis or even a dedifferentiation with a reduced α- and β-cell mass remains to be elucidated. Together, these observations suggest that islet cholinergic signaling through α7 nAChR may play an essential role in mechanisms controlling α- and β-cell mass.

The absence of α7 nAChR in pregnant female mice could promote insulin resistance, which could contribute to higher levels of blood glucose (Figure 5j). It is now well accepted that fetuses in response to a mild hyperglycemic intrauterine environment exhibit compensatory increases in β-cell mass [52], pancreatic insulin content [52] and hyperinsulinemia [52]. Moreover, expression of IRS-2 [53], pancreatic IGF-1 [54], and islet IGF-1R [54] are also increased. At this stage, insulin and IGF-1 act as growth factors on fetuses resulting in macrosomia [36]. Since the intrauterine environment of α7^−/−^ mothers is mildly hyperglycemic (Figure 5i,j), it is not surprising that β-cell mass is increased in α7^−/−^ fetuses, which are macrosomic (Figure 5b,g). This suggests that remodeling of β-cell mass in α7^−/−^ fetuses is likely attributable to mild hyperglycemic intrauterine environment, which triggered mechanisms of compensatory β-cell expansion in spite of the lack of α7 nAChR. Thus, we conclude that changes in β-cell mass observed in α7^−/−^ fetuses are likely attributable to both mild high glycemia *in utero* and the absence of α7 nAChR. Another possible explanation is that the lack of α7^−/−^ nAChR in β-cells prevented their expansion in response to mild hyerglycemic intrauterine environment. To compensate this defective proliferation, α-cells could be reprogrammed into β cells, in accordance with what has been reported by previous experimental studies [55,56].

In humans, the gradual decline of functional β-cell mass rises glycemia and ultimately leads to T2D. Here, we observed that the decline of α7^−/−^ β-cell mass was paralleled by progressive increase in glucose levels (in 1-week-old mice) leading to a chronic mild high glycemia state in 10- to 25-week-old mice. Importantly, insulin sensitivity was not significantly different between 10-week-old α7^−/−^ and WT control mice. Ten-week-old α7^−/−^ mice displayed a tendency higher insulin sensitivity. Consequently, blood glucose levels increase at 10 weeks of age cannot be due to insulin resistance but rather to a reduction in α7^−/−^ islet mass. Our findings in vivo and in vitro showed that α7^−/−^ islets increased basal insulin secretion, which is likely to maintain a normal GSIS, thus revealing a compensatory α7^−/−^ β-cell adaptation to prevent or delay the onset of T2D [57]. Moreover, mRNA expression of GLUT2 glucose transporter in pancreatic islets from 10-week-old α7^−/−^ mice was significantly increased (Figure 1g), which is another compensatory α7^−/−^ β-cell adaptation likely to maintain a normal GSIS. Therefore, implementation of these compensatory pathways, in an attempt to improve glucose homeostasis, resulted in chronic mild high glycemia not exceeding 200 mg/dL in 10-week-old α7^−/−^ mice, which can be considered as a pre-diabetic state in mice [58]. Nevertheless, we observed that the fold change in insulin secretion, in response to 16.7mM glucose, tended to decrease in α7^−/−^ islets from 10-week-old mice, reflecting the onset of insulin secretion dysfunction from β-cells to respond to elevated glucose levels. This is supported by the HOMA-β index, which tends to be lower in 10-week-old α7^−/−^ mice (Figure 1k). These data are consistent with previous studies showing that β-cell chronic exposure to mild high glycemia may induce their dysfunction over time [59,60,61].

Insulin resistance is a significant metabolic disorder in both pre-diabetic and diabetic patients [62]. Interestingly, it has been reported that pharmacological activation of α7 nAChR enhances insulin sensitivity [23]. We report here that the lack of α7 nAChR leads to insulin resistance in 25-week-old mice as shown by markedly reduced insulin-stimulated glucose uptake in EDL skeletal muscle, supporting α7 nAChR involvement in insulin sensitivity. As skeletal muscle insulin resistance acts a central role in the pathogenesis of T2D [63,64], it is possible that α7^−/−^ mice with age or exposed to high fat diet may develop T2D.

Our study suggests that weight gain observed here may be mediated by the lack of α7 nAChR in peripheral tissues (Figure 1a–c). Indeed, the lack of α7 nAChR significantly increased fat mass gain and food intake, whereas pharmacological activation of α7 nAChR in *db*/*db* obese mice decreased weight gain and food intake [21]. Therefore, α7 nAChR activation by specific agonists or by nicotine, an exogenous ligand, could potentially improve metabolic disorders such as obesity and diabetes. Thus, nicotine treatment increased insulin sensitivity in obese rats [65], improved glucose homeostasis in *db/db* obese mice and reduced cytokine and NEFA levels in WT mice [22]. Moreover, α7 nAChR activation by a specific agonist in *db*/*db* obese mice decreased weight gain and food intake. Besides, previous studies support that chronic inflammation, particularly in adipose tissue, may be a molecular mechanism involved in obesity [66,67]. Interestingly, pharmacological activation of α7 nAChR in *db*/*db* obese mice suppressed inflammation in adipose tissue by stimulating the cholinergic anti-inflammatory pathway [21,22]. On the other hand, we found that insulin-resistant α7^−/−^ mice exhibit an excessive inflammation in adipose tissue (Figure 7a), which is probably in response to enhanced plasma NEFA levels (Figure 3f), which are known factors causing inflammation and insulin resistance [22]. Together, these observations support the idea that α7 nAChR expressed in peripheral tissues is involved in body weight regulation. However, we cannot rule out the possibility that metabolic effects observed are also attributable to the lack of α7 nAChR in hypothalamic nuclei, which regulate food intake [68]. Indeed, it is believed that activation of hypothalamic α7 nAChRs trigger mechanisms for appetite suppression [69], leading to a reduced food intake [70]. Thus, we believe that α7 nAChRs expressed in peripheral tissues and hypothalamic nuclei are essential players involved in molecular mechanisms related to obesity.

α7 nAChR is widely expressed in both peripheral tissues (Figure 1) and hypothalamus [71]. Therefore, one inherent weakness of our study is the use of a constitutive KO mouse model, which does not allow us to pinpoint precisely the anatomically relevant site or sites related to metabolic disorders observed in α7^−/−^ mice. In addition, results from our mouse model are difficult to extrapolate to human because the total lack of α7 nAChR has never been reported. On the other hand, dupα7, a hybrid gene (*CHRFAM7A*), was identified, resulting from the fusion of a partial duplication of *CHRNA7* gene with the novel *FAM7A* gene [72]. It has been reported that dupα7 exerts a negative effect on α7 nAChR activity when co-expressed in oocytes [73], but to date there is no experimental study reporting the functional role of dupα7 in eukaryote cells. It is possible that moderate downregulation of α7 nAChR may exert only a moderate impact on glucose homeostasis in human compared to mouse. However, our study suggests that α7^−/−^ mouse may be a suitable model for the typical human late onset obesity when fed a standard chow. Indeed, this mouse model mimics human obesity because it is characterized by a slow and gradual mass fat accumulation over the time (Figure 6). Interestingly, α7 nAChR expression levels were significantly lower in subcutaneous adipocyte tissue of obese subjects compared with that of normal-weight subjects [20]. Its expression was also downregulated at protein level in these obese patients [20]. Moreover, α7^−/−^ mouse also shows diabetes-like syndromes, such as a reduction in β-cell mass, chronic mild high glycemia, glucose intolerance, and insulin resistance; the metabolic phenotypes greatly resembling those observed in pre-diabetic and mild diabetic patients as well as in heavy smokers [12]. It is now believed that the overall effect of sustained smoking may result in nAChR desensitization and that chronic smoking maintains this desensitization [74]. The α7 nAChR has been shown to be particularly susceptible to desensitization [75,76]. Therefore, α7^−/−^ mouse may be considered as a model mimicking heavy smokers. Thus, inactivation of peripheral and central α7 nAChRs, as a consequence of either down-regulated expression or desensitization by prolonged nicotine exposure in smokers [77], could alter their glucose homeostasis, as observed in α7^−/−^ mice. Thus, our study also unveils the α7^−/−^ mouse model as a powerful tool to study the mechanisms leading to impaired glucose homeostasis related to smoking.

## 5. Conclusions

In summary, our results show that the lack of α7 nAChR is associated with a chronic mild high glycemia, glucose intolerance, as well as reduction in α- and β-cell mass in young male mice. Despite a deficit in β-cell mass, these male mice display a normal GSIS and tend to have better insulin sensitivity compared with control male mice, likely to compensate the deficit in β-cells. As the mice age, they develop insulin resistance, increase their fasting insulin secretion and accumulate more fat mass, thus emphasizing their fasting glycemia. Our study highlights a novel and essential role of α7 nAChR in glucose homeostasis in mice and identify its total absence as a predisposing factor in the pathogenesis of chronic high glycemia. Our findings also suggest that α7 nAChR could be a new therapeutic target for strategies to treat diabetic patients and smokers exhibiting an impaired glucose homeostasis.

## Figures and Tables

**Table 1:** Distribution of experiments by set of animals.

	*Set 1*	*Set 2*	*Set 3*	*Set 4*	*Set 5*	*Set 6*	*Set 7*	*Set 8*
*Body weight*	X	X	X	X				X
*Pancreas weight*		X		X				X
*Non-fasting glycemia*	X		X	X				X
*IPGTT, GSIS, ITT*				X				X
*IPPTT*								X
*2DG Uptake*								X
*Food intake*					X	X	X	X
*EchoMRI*						X	X	X
*β- and α- cell mass*		X		X				
*Insulin content*				X				
*Static incubation*				X				
*Hepatic glycogen content*								X
*Plasma NEFA*								X
*qPCR*		X		X				X

**Table 2:** Genes analyzed by Real-Time PCR and primer sequences.

Gene	Polarity	Sequence (5′ to 3′)	Genebank Number
M3 mAChR	Forward	ACCAGGCCACTCACTTACCG	12671
	Reverse	CCCAAGCCAGACCAACCCTCATCACAC	
α3 nicotinic subunit	Forward	ATATGAAAGCACAGAATGTAGCCA	110,834
	Reverse	TGCACACCAGGATGAAAACCCA	
α5 nicotinic subunit	Forward	TGATCAAGCATTCAATCTATGGGT	110,835
	Reverse	CACAAAAGCCATTCCATATTGCC	
α6 nicotinic subunit	Forward	CGAGAGTGTTCAATTCATAGCAGA	11,440
	Reverse	GACTTTCCTGTGTTCCCAAGCA	
α7 nicotinic subunit	Forward	TATCCCTGTAGCTGTCGGTC	11,441
	Reverse	CCTGCCGAGTACAATGATATGCC	
β2 nicotinic subunit	Forward	TTGAAATCTACTAAGCCCCACAAC	11,444
	Reverse	TTCCCTGCTTCAAAACTTTTCCC	
β4 nicotinic subunit	Forward	CCTTGCCCATTAAAGTCTATGCTC	11,438
	Reverse	CACACACTCCGACCCTTG	
BAD	Forward	TTCCAGATCCCAGAGTTTGAGCC	12,015
	Reverse	CTGTAGCACTAGCGTCTTCC	
BAX	Forward	CACCAAGAAGCTGAGCGAGT	12,028
	Reverse	CCCCAGTTGAAGTTGCCATCA	
BCL2	Forward	AGCGGATATACCTTTCTTACCCT	12,043
	Reverse	CTTGATTCTGGTGTTTCCCCGTTG	
CXCL1	Forward	CCGAAGTCATAGCCACACTCA	14,825
	Reverse	TCAGAAGCCAGCGTTCACCA	
F4/80	Forward	CCAAGCCTATTATCTATACCCTCC	13,733
	Reverse	CCAAGTTTGCCATCCGGTT	
GLUT 2	Forward	TCTGTTACCTTTCCTCGGCTTC	20,526
	Reverse	ACAGCTAAGAACAACTCAAGCATT	
IGF 2	Forward	ACCCAGTGTTAATATGCCTCGT	16,002
	Reverse	AAGACAGTAGGGAAGAGACAAGA	
IL-1β	Forward	CCTGTGTTTTCCTCCTTGCCT	16,176
	Reverse	TCTCAGCTTCAATGAAAGACCTC	
IL-6	Forward	GAGACTTCCATCCAGTTGCC	16,193
	Reverse	AAGTAGGGAAGGCCGTGGTT	
IL-15	Forward	CTTGCTTATTTTTAATTTATTGCT	16,168
	Reverse	AAAATATCCATACAACTTTATTCCAC	
TLR4	Forward	CAGCAGGTGGAATTGTATCGC	21,898
	Reverse	TGCTCAGGATTCGAGGCTTT	
TNFα	Forward	AGCACAGAAAGCATGATCCG	21,926
	Reverse	ACCCCGAAGTTCAGTAGACAG	
Cyclophilin A	Forward	CGTGGCCAACGATAAGAAGAA	268,373
	Reverse	GTCTCCACCCTGGATCATGAA	
HPRT	Forward	TCCTCCTCAGACCGCTTTT	15,452
	Reverse	CCTGGTTCATCATCGCTAATC	
RPL19	Forward	GGGCAGGCATATGGGCATA	19,921
	Reverse	GGCGGTCAATCTTCTTGGATT	
TBP	Forward	GGGGAGCTGTGATGTGAAGT	21,374
	Reverse	CCAGGAAATAATTCTGGCTCA	

## Abbrevation

AOC	Area Over Curve
AUC	Aera Under Curve
Chrna7	Cholinergic receptor nicotinic alpha7
Cyclo A	Cyclophilin A
EDL	Extensor Digitorum Longus
GSIS	glucose-stimulated insulin secretion
GTT	Glucose tolerance test
HPRT	Hypoxanthine phosphoribosyl transferase
ITT	Insulin tolerance test
IGF-1	Insulin-like growth factor-1
IGF-1R	Insulin-like growth factor-1 receptor
IGF-2	Insulin-like growth factor-2
IRS 2	Insulin receptor substrate 2
mAChRs	muscarinic acetylcholine receptors
M-MLV	Moloney-murine leukemia virus
nAChR	nicotinic acetylcholine receptor
NEFAs	Non-esterified fatty acids
PTT	Pyruvate tolerance test
RPL19	Ribosomal protein L19
TBP	TATA-box binding protein
T1D	type 1 diabetes
T2D	type 2 diabetes
WT	Wild-Type

## References

[1] Guariguata L., Whiting D.R., Hambleton I.R., Beagley J., Linnenkamp U., Shaw J. (2014). Global estimates of diabetes prevalence for 2013 and projections for 2035. Diabetes Res. Clin. Pract..

[2] DeFronzo R.A. (2004). Pathogenesis of type 2 diabetes mellitus. Med. Clin. N. Am..

[3] Sowers J.R., Frohlich E.D. (2004). Insulin and insulin resistance: Impact on blood pressure and cardiovascular disease. Med. Clin. N. Am..

[4] Halban P.A., Polonsky K.S., Bowden N.W., Hawkins M.A., Ling C., Mather K.J., Powers A.C., Rhodes C.J., Sussel L., Weir G.C. (2014). β-Cell Failure in Type 2 Diabetes: Postulated Mechanisms and Prospects for Prevention and Treatment. J. Clin. Endocrinol. Metab..

[5] Ahrén B. (2000). Autonomic regulation of islet hormone secretion—Implications for health and disease. Diabetologia.

[6] Lausier J., Diaz W.C., Roskens V., LaRock K., Herzer K., Fong C.G., Latour M.G., Peshavaria M., Jetton T.L. (2010). Vagal control of pancreatic ß-cell proliferation. Am. J. Physiol. Metab..

[7] Guo Y., Traurig M., Ma L., Kobes S., Harper I., Infante A.M., Bogardus C., Baier L.J., Prochazka M. (2006). CHRM3 Gene Variation Is Associated With Decreased Acute Insulin Secretion and Increased Risk for Early-Onset Type 2 Diabetes in Pima Indians. Diabetes.

[8] Gilon P. (2001). Mechanisms and Physiological Significance of the Cholinergic Control of Pancreatic -Cell Function. Endocr. Rev..

[9] Ejiri K., Taniguchi H., Ishihara K., Hara Y., Baba S. (1990). Possible involvement of cholinergic nicotinic receptor in insulin release from isolated rat islets. Diabetes Res. Clin. Pract..

[10] Yoshikawa H., Hellström-Lindahl E., Grill V. (2005). Evidence for functional nicotinic receptors on pancreatic β cells. Metabolism.

[11] Ohtani M., Oka T., Badyuk M., Xiao Y., Kellar K.J., Daly J.W. (2005). Mouse β-TC6 Insulinoma Cells: High Expression of Functional α3β4 Nicotinic Receptors Mediating Membrane Potential, Intracellular Calcium, and Insulin Release. Mol. Pharmacol..

[12] Maddatu J., Anderson-Baucum E., Evans-Molina C. (2017). Smoking and the risk of type 2 diabetes. Transl. Res..

[13] Ganic E., Singh T., Luan C., Fadista J., Johansson J.K., Cyphert H.A., Bennet H., Storm P., Prost G., Ahlenius H. (2016). MafA-Controlled Nicotinic Receptor Expression Is Essential for Insulin Secretion and Is Impaired in Patients with Type 2 Diabetes. Cell Rep..

[14] Changeux J.-P. (2012). The Nicotinic Acetylcholine Receptor: The Founding Father of the Pentameric Ligand-gated Ion Channel Superfamily. J. Biol. Chem..

[15] Steinbach J.H. (2007). Mechanism of Action of the Nicotinic Acetylcholine Receptor. Ciba Found. Symp. Bilharz..

[16] Séguéla P., Wadiche J., Dineley-Miller K., Dani J., Patrick J. (1993). Molecular cloning, functional properties, and distribution of rat brain alpha 7: A nicotinic cation channel highly permeable to calcium. J. Neurosci..

[17] Maouche K., Polette M., Jolly T., Medjber K., Cloëz-Tayarani I., Changeux J.-P., Burlet H., Terryn C., Coraux C., Zahm J.-M. (2009). α7 Nicotinic Acetylcholine Receptor Regulates Airway Epithelium Differentiation by Controlling Basal Cell Proliferation. Am. J. Pathol..

[18] Catassi A., Servent D., Paleari L., Cesario A., Russo P. (2008). Multiple roles of nicotine on cell proliferation and inhibition of apoptosis: Implications on lung carcinogenesis. Mutat. Res. Mutat. Res..

[19] Wang H., Yu M., Ochani M., Amella C.A., Tanovic M., Susarla S., Li J.H., Wang H., Yang H., Ulloa L. (2002). Nicotinic acetylcholine receptor α7 subunit is an essential regulator of inflammation. Nature.

[20] Cancello R., Zulian A., Maestrini S., Mencarelli M., Della Barba A., Invitti C., Liuzzi A., Di Blasio A.M. (2012). The nicotinic acetylcholine receptor α7 in subcutaneous mature adipocytes: Downregulation in human obesity and modulation by diet-induced weight loss. Int. J. Obes..

[21] Marrero M.B., Lucas R., Salet C., Hauser T.A., Mazurov A., Lippiello P.M., Bencherif M. (2009). An α7 Nicotinic Acetylcholine Receptor-Selective Agonist Reduces Weight Gain and Metabolic Changes in a Mouse Model of Diabetes. J. Pharmacol. Exp. Ther..

[22] Wang X., Yang Z., Xue B., Shi H. (2011). Activation of the cholinergic antiinflammatory pathway ameliorates obesity-induced inflammation and insulin resistance. Endocrinology.

[23] Xu T.-Y., Guo L.-L., Wang P., Song J., Le Y., Viollet B., Miao C.-Y. (2012). Chronic Exposure to Nicotine Enhances Insulin Sensitivity through α7 Nicotinic Acetylcholine Receptor-STAT3 Pathway. PLoS ONE.

[24] Somm E., Guérardel A., Maouche K., Toulotte A., Veyrat-Durebex C., Rohner-Jeanrenaud F., Maskos U., Hüppi P.S., Schwitzgebel V.M. (2014). Concomitant alpha7 and beta2 nicotinic AChR subunit deficiency leads to impaired energy homeostasis and increased physical activity in mice. Mol. Genet. Metab..

[25] Lerat H., Imache M.R., Polyte J., Gaudin A., Mercey M., Donati F., Baudesson C., Higgs M.R., Picard A., Magnan C. (2017). Hepatitis C virus induces a prediabetic state by directly impairing hepatic glucose metabolism in mice. J. Biol. Chem..

[26] Movassat J., Saulnier C., Serradas P., Portha B. (1997). Impaired development of pancreatic beta-cell mass is a primary event during the progression to diabetes in the GK rat. Diabetologia.

[27] Figeac F., Ilias A., Bailbé D., Portha B., Movassat J. (2012). Local In Vivo GSK3β Knockdown Promotes Pancreatic β Cell and Acinar Cell Regeneration in 90% Pancreatectomized Rat. Mol. Ther..

[28] Cruciani-Guglielmacci C., Bellini L., Denom J., Oshima M., Fernandez N., Normandie-Levi P., Berney X.P., Kassis N., Rouch C., Dairou J. (2017). Molecular phenotyping of multiple mouse strains under metabolic challenge uncovers a role for Elovl2 in glucose-induced insulin secretion. Mol. Metab..

[29] Bansal N. (2015). Prediabetes diagnosis and treatment: A review. World J. Diabetes.

[30] Kergoat M., Guerre-Millo M., Lauva M., Portha B. (1991). Increased insulin action in rats with mild insulin deficiency induced by neonatal streptozotocin. Am. J. Physiol. Metab..

[31] Randle P.J. (1998). Regulatory interactions between lipids and carbohydrates: The glucose fatty acid cycle after 35 years. Diabetes Metab. Rev..

[32] Nuutila P., Koivisto V.A., Knuuti J., Ruotsalainen U., Teräs M., Haaparanta M., Bergman J., Solin O., Voipio-Pulkki L.M., Wegelius U. (1992). Glucose-free fatty acid cycle operates in human heart and skeletal muscle in vivo. J. Clin. Investig..

[33] Lam T.K.T., Van De Werve G., Giacca A. (2003). Free fatty acids increase basal hepatic glucose production and induce hepatic insulin resistance at different sites. Am. J. Physiol. Metab..

[34] Stingl H., Krššák M., Krebs M., Bischof M.G., Nowotny P., Fürnsinn C., Shulman G.I., Waldhäusl W., Roden M. (2001). Lipid-dependent control of hepatic glycogen stores in healthy humans. Diabetologia.

[35] Perseghin G., Petersen K., Shulman G.I. (2003). Cellular mechanism of insulin resistance: Potential links with inflammation. Int. J. Obes..

[36] Portha B., Chavey A., Movassat J. (2011). Early-Life Origins of Type 2 Diabetes: Fetal Programming of the Beta-Cell Mass. Exp. Diabetes Res..

[37] Lumeng C.N., Saltiel A.R. (2011). Inflammatory links between obesity and metabolic disease. J. Clin. Investig..

[38] Hotamisligil G.S. (2006). Inflammation and metabolic disorders. Nature.

[39] Ramanathan S., Lacraz G., Donates Y.C., Mayhue M., Langois M.F., Pleszczynski M.R., Ilangumaran S. (2014). Interleukin-15 in the Regulation of Obesity and Fatty Liver Disease. J. Clin. Exp. Hepatol..

[40] Nunemaker C.S., Chung H.G., Verrilli G.M., Corbin K.L., Upadhye A., Sharma P.R. (2014). Increased serum CXCL1 and CXCL5 are linked to obesity, hyperglycemia, and impaired islet function. J. Endocrinol..

[41] Wajchenberg B.L. (2007). β-Cell Failure in Diabetes and Preservation by Clinical Treatment. Endocr. Rev..

[42] Pick A., Clark J., Kubstrup C., Levisetti M., Pugh W., Bonner-Weir S., Polonsky K.S. (1998). Role of apoptosis in failure of beta-cell mass compensation for insulin resistance and beta-cell defects in the male Zucker diabetic fatty rat. Diabetes.

[43] Woods S.C., Porte D. (1974). Neural control of the endocrine pancreas. Physiol. Rev..

[44] Edvell A., Lindström P. (1998). Vagotomy in young obese hyperglycemic mice: Effects on syndrome development and islet proliferation. Am. J. Physiol. Content.

[45] Yamamoto J., Imai J., Izumi T., Takahashi H., Kawana Y., Takahashi K., Kodama S., Kaneko K., Gao J., Uno K. (2017). Neuronal signals regulate obesity induced β-cell proliferation by FoxM1 dependent mechanism. Nat. Commun..

[46] Gupta D., Lacayo A.A., Greene S.M., Leahy J.L., Jetton T.L. (2018). β-Cell mass restoration by α7 nicotinic acetylcholine receptor activation. J. Biol. Chem..

[47] Fujimoto K., Polonsky K.S. (2009). Pdx1 and other factors that regulate pancreatic beta-cell survival. Diabetes Obes. Metab..

[48] Duttaroy A., Zimliki C.L., Gautam D., Cui Y., Mears D., Wess J. (2004). Muscarinic stimulation of pancreatic insulin and glucagon release is abolished in m3 muscarinic acetylcholine receptor-deficient mice. Diabetes.

[49] Gautam D., Han S.-J., Hamdan F.F., Jeon J., Li B., Li J.H., Cui Y., Mears D., Lu H., Deng C. (2006). A critical role for β cell M3 muscarinic acetylcholine receptors in regulating insulin release and blood glucose homeostasis in vivo. Cell Metab..

[50] Devedjian J.-C., George M., Casellas A., Pujol A., Visa J., Pelegrin M., Gros L., Bosch F. (2000). Transgenic mice overexpressing insulin-like growth factor-II in β cells develop type 2 diabetes. J. Clin. Investig..

[51] Petrik J., Reusens B., Arany E., Remacle C., Coelho C., Hoet J.J., Hill D.J. (1999). A Low Protein Diet Alters the Balance of Islet Cell Replication and Apoptosis in the Fetal and Neonatal Rat and Is Associated with a Reduced Pancreatic Expression of Insulin-Like Growth Factor-II1. Endocrinology.

[52] Alvarez C. (1997). Contrasted Impact of Maternal Rat Food Restriction on the Fetal Endocrine Pancreas. Endocrinology.

[53] Fernandez E., Martín M.A., Fajardo S., Escrivá F., Alvarez C. (2007). Increased IRS-2 content and activation of IGF-I pathway contribute to enhance β-cell mass in fetuses from undernourished pregnant rats. Am. J. Physiol. Metab..

[54] Martín M.A., Serradas P., Ramos S., Fernandez E., Goya L., Gangnerau M.N., Lacorne M., Pascual-Leone A.M., Escrivá F., Portha B. (2005). Protein-Caloric Food Restriction Affects Insulin-Like Growth Factor System in Fetal Wistar Rat. Endocrinology.

[55] Thorel F., Nepote V., Avril I., Kohno K., Desgraz R., Chera S., Herrera P.L. (2010). Conversion of adult pancreatic α-cells to β-cells after extreme β-cell loss. Nature.

[56] Yang Y.-P., Thorel F., Boyer D.F., Herrera P.L., Wright C.V.E. (2011). Context-specific α-to-β-cell reprogramming by forced Pdx1 expression. Genes Dev..

[57] Weir G.C., Bonner-Weir S. (2004). Five of stages of evolving β-cell dysfunction during progression to diabetes. Diabetes.

[58] King A.J.F. (2012). The use of animal models in diabetes research. Br. J. Pharmacol..

[59] Bonner-Weir S., Trent D.F., Honey R.N., Weir G.C. (1981). Responses of neonatal rat islets to streptozotocin: Limited B-cell regeneration and hyperglycemia. Diabetes.

[60] Leahy J.L., Cooper H.E., Weir G.C. (1987). Impaired Insulin Secretion Associated With Near Normoglycemia: Study in Normal Rats With 96-h In Vivo Glucose Infusions. Diabetes.

[61] Laury M.C., Takao F., Bailbé D., Penicaud L., Portha B., Picon L., Ktorza G.A. (1991). Differential Effects of Prolonged Hyperglycemia on in Vivo and in Vitro Insulin Secretion in Rats. Endocrinology.

[62] Chen M., Porte D. (1976). The Effect of Rate and Dose of Glucose Infusion on the Acute Insulin Response in Man. J. Clin. Endocrinol. Metab..

[63] Thiebaud D., Jacot E., DeFronzo R.A., Maeder E., Jequier E., Felber J.-P. (1982). The Effect of Graded Doses of Insulin on Total Glucose Uptake, Glucose Oxidation, and Glucose Storage in Man. Diabetes.

[64] Lillioja S., Mott D.M., Spraul M., Ferraro R., Foley J.E., Ravussin E., Knowler W.C., Bennett P.H., Bogardus C. (1993). Insulin Resistance and Insulin Secretory Dysfunction as Precursors of Non-Insulin-Dependent Diabetes Mellitus: Prospective Studies of Pima Indians. N. Engl. J. Med..

[65] Liu R., Kurose T., Matsukura S. (2001). Oral nicotine administration decreases tumor necrosis factor-alpha expression in fat tissues in obese rats. Metabolism.

[66] Hotamisligil G., Shargill N., Spiegelman B. (1993). Adipose expression of tumor necrosis factor-alpha: Direct role in obesity-linked insulin resistance. Science.

[67] Uysal K.T., Wiesbrock S.M., Hotamisligil G.S. (1998). Functional Analysis of Tumor Necrosis Factor (TNF) Receptors in TNF-α-Mediated Insulin Resistance in Genetic Obesity. Endocrinology.

[68] Schwartz M.W., Woods S.C., Porte D., Seeley R.J., Baskin D.G. (2000). Central nervous system control of food intake. Nature.

[69] McFadden K.L., Cornier M.-A., Tregellas J.R. (2014). The role of alpha-7 nicotinic receptors in food intake behaviors. Front. Psychol..

[70] Jo Y.-H., Talmage D., Role L.W. (2002). Nicotinic receptor-mediated effects on appetite and food intake. J. Neurobiol..

[71] Souza A., Souza C.M., Amaral C.L., Lemes S.F., Santucci L.F., Milanski M., Torsoni A.S., Torsoni M.A. (2019). Short-Term High-Fat Diet Consumption Reduces Hypothalamic Expression of the Nicotinic Acetylcholine Receptor α7 Subunit (α7nAChR) and Affects the Anti-inflammatory Response in a Mouse Model of Sepsis. Front. Immunol..

[72] Riley B., Williamson M., Collier D., Wilkie H., Makoff A. (2002). A 3-Mb Map of a Large Segmental Duplication Overlapping the α7-Nicotinic Acetylcholine Receptor Gene (CHRNA7) at Human 15q13–q14. Genomics.

[73] De Lucas-Cerrillo A.M., Maldifassi M.C., Arnalich F., Renart J., Atienza G., Serantes R., Cruces J., Pacheco A.S., Andrés-Mateos E., Montiel C. (2010). Function of Partially Duplicated Human α7 Nicotinic Receptor Subunit CHRFAM7A Gene. J. Biol. Chem..

[74] Brody A.L., Mandelkern M.A., London E.D., Olmstead R.E., Farahi J., Scheibal D., Jou J., Allen V., Tiongson E., Chefer S.I. (2006). Cigarette Smoking Saturates Brain α4β2 Nicotinic Acetylcholine Receptors. Arch. Gen. Psychiatry.

[75] Corringer P.-J., Bertrand S., Bohler S., Edelstein S.J., Changeux J.-P., Bertrand D. (1998). Critical Elements Determining Diversity in Agonist Binding and Desensitization of Neuronal Nicotinic Acetylcholine Receptors. J. Neurosci..

[76] Olale F., Gerzanich V., Kuryatov A., Wang F., Lindstrom J. (1997). Chronic nicotine exposure differentially affects the function of human α3, α4, and α7 neuronal nicotinic receptor subtypes. J. Pharmacol. Exp. Ther..

[77] Giniatullin R., Nistri A., Yakel J.L. (2005). Desensitization of nicotinic ACh receptors: Shaping cholinergic signaling. Trends Neurosci..

